# Towards Maintenance 5.0: Resilience-Based Maintenance in AI-Driven Sustainable and Human-Centric Industrial Systems

**DOI:** 10.3390/s25165100

**Published:** 2025-08-16

**Authors:** Lech Bukowski, Sylwia Werbinska-Wojciechowska

**Affiliations:** 1 Faculty of Applied Sciences, WSB University, 1c Zygmunta Cieplaka Street, 41-300 Dabrowa Gornicza, Poland; lbukowski@wsb.edu.pl; 2 Faculty of Mechanical Engineering, Wroclaw University of Science and Technology, Wyspianskiego 27, 50-370 Wroclaw, Poland

**Keywords:** resilience-based maintenance, Maintenance 5.0, Industry 5.0, artificial intelligence, sustainable maintenance, human-centric systems, digital twin, predictive maintenance, PRISMA review, maintenance strategies

## Abstract

Industry 5.0 introduces a new paradigm where digital technologies support sustainable and human-centric industrial development. Within this context, resilience-based maintenance (RBM) emerges as a forward-looking maintenance strategy focused on system adaptability, fault tolerance, and recovery capacity under uncertainty. This article presents a systematic literature review (SLR) on RBM in the context of Maintenance 5.0. The review follows the PRISMA methodology and incorporates bibliometric and content-based analyses of selected publications. Key findings highlight the integration of AI methods, such as machine learning and digital twins, in enhancing system resilience. The results demonstrate how RBM aligns with the pillars of Industry 5.0, sustainability, and human-centricity, by reducing resource consumption and improving human–machine interaction. Research gaps are identified in AI explainability, sector-specific implementation, and ergonomic integration. The article concludes by outlining directions for developing Maintenance 5.0 as a strategic concept for resilient, intelligent, and inclusive industrial systems.

## 1. Introduction

The dynamic evolution of industrial systems has been shaped by the shift from Industry 4.0, focused on digitalization, automation, and connectivity, to the emerging paradigm of Industry 5.0, which integrates technological progress with sustainability, resilience, and human-centricity [1,2]. Rather than replacing Industry 4.0, Industry 5.0 builds on its foundations, refocusing priorities toward well-being, environmental responsibility, and socio-technical balance.

In this evolving context, traditional maintenance approaches, focused on minimizing downtime or maximizing reliability through reactive, preventive, or predictive strategies, are no longer sufficient. Maintenance must evolve toward a more adaptive and systemic form capable of supporting resilience in complex, volatile environments [3,4,5].

One promising approach is resilience-based maintenance (RBM), which emphasizes a system’s ability to withstand disturbances, recover operational capabilities, and learn from disruptions [6]. RBM incorporates adaptability, redundancy, and self-organization principles beyond fault prediction and condition monitoring [7]. The growing importance of AI in this context is central—artificial intelligence (AI), machine learning (ML), and digital twins (DTs) offer new capabilities to model, predict, and manage maintenance under uncertainty [8], directly supporting the development of RBM.

Despite the increasing attention to sustainable maintenance in recent literature, most existing systematic reviews remain fragmented or domain-specific, lacking a comprehensive framework that bridges resilience, sustainability, and AI integration. For example, Campos and Simon [9] discuss the insertion of sustainability concepts in maintenance strategies, while Zhao et al. [10] and Hallioui et al. [11] review sustainable maintenance practices for specific systems or approaches such as sustainable total productive maintenance (STPM). Vasić et al. [12] conducted an umbrella review on sustainability adoption criteria, and Madreiter et al. [13] identified technology drivers enabling positive sustainable impact in manufacturing. However, none of these works offer a holistic integration of sustainability with resilience and AI-based decision-making under the Industry 5.0 paradigm. Therefore, while sustainability is often addressed in separate maintenance strategies [1,9,10,11,14,15], integrated perspectives combining it with resilience and digital intelligence remain rare.

Similarly, recent reviews have explored predictive maintenance (PdM) (see, e.g., [16,17,18,19,20]), reliability-centered maintenance (RCM) (see, e.g., [14,21,22]), or AI applications in diagnostics (see, e.g., [23,24,25,26]). Analyses of the evolution of maintenance practices from Industry 4.0 to Industry 5.0 context are presented, e.g., in [15,27,28]. In addition, recent developments in the area of Maintenance 5.0 concept implementation challenges and possibilities are discussed, e.g., in [29,30]. However, they rarely connect technological advancements with the societal goals emphasized in Industry 5.0.

Some recent works have begun to address broader conceptual frameworks, such as Maintenance 5.0 or smart maintenance, in relation to sustainability and resilience. For example, Jasiulewicz-Kaczmarek et al. [31,32] explored how Maintenance 4.0 technologies support sustainability goals, while Werbińska-Wojciechowska and Winiarska [3] provided a bibliometric and systematic analysis of smart maintenance performance in Industry 4.0 environments. Saihi et al. [33] presented a modeling-based review of sustainable maintenance, identifying key indicators and trade-offs. Bastas [34] contributed to understanding sustainability-oriented production technologies without directly addressing maintenance. In addition, sustainable maintenance in the context of Industry 4.0 concept implementation is also discussed, e.g., in [35,36,37]. The aspects of resilience-based maintenance are reviewed, e.g., in [7,38].

It is worth taking note that there are also works on human-centricity in maintenance management. For example, the authors in [39] focused on the Operator 4.0 concept, analyzing the occupational risks workers face and the proposed solutions to support them by leveraging the key enabling technologies of Industry 4.0. This problem is continued in [40], where the authors provided a literature review on human-centricity in Industry 5.0. Socio-economic dimensions are also reviewed in [41]. Recent reviews from 2024 to 2025 have further highlighted the growing focus on digital sustainable maintenance [42], prescriptive maintenance [43,44], and umbrella reviews of reviews [45,46], confirming that while the literature continues to expand rapidly few studies offer an integrative view that combines resilience, sustainability, and human-centricity with AI-based tools under the unified framework of Maintenance 5.0. However, these existing contributions offer only fragmented perspectives and do not provide a structured approach to integrating resilience, sustainability, and human-centricity into maintenance strategies. Moreover, they often lack a dedicated focus on the domain of industrial maintenance, which is a critical enabler of operational continuity, especially under disruptive conditions. There is a notable gap in the literature regarding linking these principles with modern AI-based tools to form a cohesive framework that aligns with the Industry 5.0 paradigm.

This article addresses this gap by introducing a structured and focused approach to Resilience-Based Maintenance in industrial maintenance management. The study’s novelty lies in its comprehensive examination of RBM as a strategic concept for ensuring operational continuity, system resilience, and sustainable development, supported by AI technologies and embedded within the Industry 5.0 vision. A comparative overview of recent review publications supporting this gap identification is presented in Appendix A.1 (Table A1). Table A1 summarizes existing review articles exploring key directions in the evolution of maintenance, particularly regarding resilience, sustainability, and AI integration. While each of these studies addresses a subset of the domain, none has provided an integrative view of RBM, sustainability, and Maintenance 5.0 under a unified framework, which this paper aims to develop and present.

In response, this study presents the results of a systematic literature review (SLR) on RBM in the context of Maintenance 5.0 and its pillars: resilience, sustainability, and human-centricity. It aims to synthesize the state of knowledge and propose a structured perspective on how RBM can support adaptability, long-term value creation, and robust decision-making using AI. This leads to the following research questions:

RQ1: What is the current state of research on resilience-based maintenance in industrial and infrastructure systems?
RQ2: Which artificial intelligence methods and tools are employed in RBM to support decision-making, adaptability, and learning?
RQ3: How is RBM aligned with the pillars of Industry 5.0, particularly sustainability and human-centricity?
RQ4: What are the key research challenges, gaps, and directions for future studies in this area?

To answer these questions, a systematic review was carried out using the PRISMA protocol [47], supported by snowball sampling to ensure inclusion of relevant but non-indexed studies [48]. The content of selected articles was analyzed using a dual approach: bibliometric analysis to identify publication trends and clusters, and content-based analysis to extract thematic insights and assess the role of AI in RBM implementation.

While RBM is applied across diverse sectors, this study deliberately adopts a cross-sectoral approach to identify transferable principles and challenges shared between industrial and infrastructure maintenance.

The structure of the paper is organized as follows: Section 2 presents the theoretical background of maintenance evolution, the concept of Maintenance 5.0, and a detailed overview of RBM. Section 3 explains the review methodology, including data sources, inclusion criteria, and analytical procedures. Section 4 summarizes the results of the SLR, combining bibliometric and content-based perspectives. Section 5 discusses the findings regarding the research questions, including identifying knowledge gaps and future development areas. Section 6 explores the implications of RBM in the context of sustainability and human-centric industrial systems. Finally, Section 7 concludes the paper by highlighting key contributions and recommendations for researchers and practitioners.

## 2. Theoretical Background

### 2.1. Evolution of Maintenance Concepts

Maintenance strategies have been profoundly transformed over the past decades, evolving from reactive, corrective practices into data-driven and intelligence-supported approaches that contribute directly to industrial systems’ performance, sustainability, and resilience [3,49]. According to IEC 60300-3-14, maintenance can be defined as the combination of all technical, administrative, and managerial actions intended to retain or restore an item to a state where it can perform its required function [50]. As industrial environments have become increasingly complex and digitalized, maintenance has shifted from a reactive necessity to a strategic, proactive, and knowledge-based function [51].

The historical development of maintenance strategies is often described through the lens of generational models [52]. The first generation was characterized by reactive maintenance, where interventions were implemented only after failure. While once acceptable in the era of simple machinery and short production cycles, this approach resulted in significant losses, safety risks, and unplanned costs. The second generation introduced preventive maintenance based on predefined schedules and usage intervals. Although more systematic, this model often led to unnecessary component replacements and did not account for system condition variability [53].

A paradigm shift occurred with the advent of condition-based and predictive maintenance strategies, marking the beginning of the third generation. These approaches leverage sensor data, diagnostic methods, and statistical models to assess the equipment’s condition and anticipate failures [54,55]. Predictive maintenance, in particular, utilizes machine learning algorithms, prognostic models, and remaining useful life (RUL) estimations to support timely and cost-effective interventions. Integrating such approaches with enterprise resource planning (ERP) and computerized maintenance management systems (CMMSs) laid the foundation for a more intelligent and responsive maintenance function [16].

The fourth generation of maintenance emerged in Industry 4.0, characterized by integrating cyber-physical systems, industrial IoT, and cloud computing. This model, known as smart maintenance, emphasizes connectivity, autonomy, and real-time decision-making. Maintenance is no longer seen as a standalone function but as part of a continuous cyber-physical production ecosystem in which machines, sensors, and algorithms interact. This generation introduced advanced tools such as digital twins, augmented reality, and edge analytics, enabling real-time diagnostics, prescriptive maintenance, and system-level optimization [56,57,58].

Today, industrial systems are entering the fifth generation of maintenance development—commonly referred to as Maintenance 5.0. This emerging paradigm aligns with the broader principles of Industry 5.0, which emphasize sustainability, resilience, and human-centricity [1]. In this context, maintenance is expected to contribute to environmental goals, system robustness, and ethical integration of automation with human work. Maintenance 5.0 integrates sustainability principles through life cycle-aware planning, resource efficiency, and environmental impact assessment. Simultaneously, it supports human well-being and agency by embedding ergonomics, transparency, and collaborative interfaces into maintenance processes. It also embraces resilience engineering, recognizing the necessity of adapting to disruptions, managing complexity, and recovering from unforeseen events [15,59].

**Figure 1:**
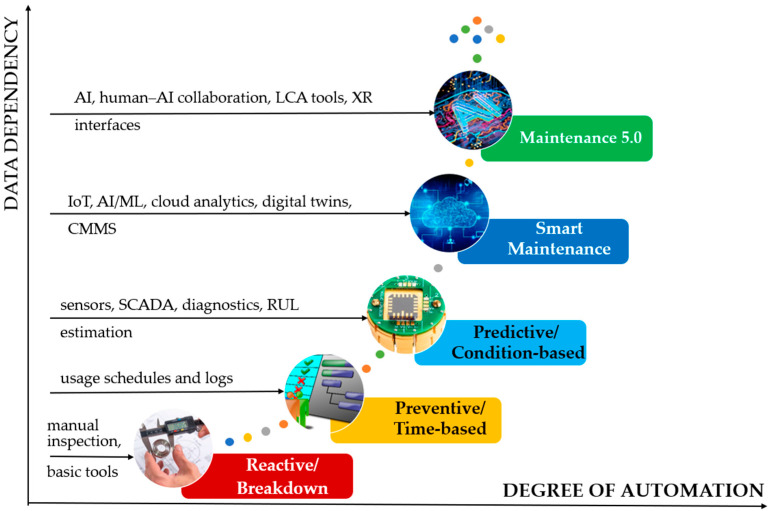
Evolution of maintenance strategies. Source: own contribution based on [3,53].

To support the clarity of this conceptual development, a graphical illustration (Figure 1) depicts the evolution of maintenance strategies from the first to the fifth generation, highlighting the main drivers, technologies, and goals associated with each stage. In addition, a comparative table (Table 1) synthesizes the distinguishing features across these generations in terms of dominant paradigms, maintenance objectives, enabling technologies, data requirements, and the role of human operators.

Recent review articles underscore this transition and identify the need for integrated maintenance strategies that combine technological innovation with resilience and sustainability. For instance, Aktef et al. [4] stress that transitioning to Maintenance 5.0 requires not only digital transformation but also embedding human-centric and sustainable values into maintenance strategies. Murtaza et al. [15] argue that predictive models must evolve into context-aware, adaptive systems that support long-term resilience.

Farsi et al. [14] suggest that reliability-centered maintenance should account for environmental and social impact, not just cost-risk tradeoffs. Kans and Campos [28] highlight organizational gaps in aligning digitalization with sustainability goals. Aktef et al. [29], through fuzzy MICMAC analysis, show that successful Maintenance 5.0 adoption depends as much on organizational readiness as on technological capability.

**Figure 2:**
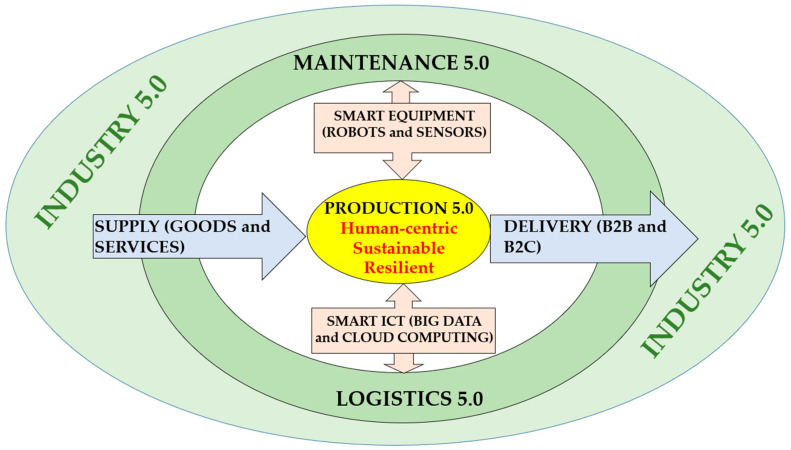
Maintenance 5.0 in the context of Industry 5.0. Source: own contribution based on [1,3].

To consolidate these insights, Figure 2 presents a conceptual framework for Maintenance 5.0, positioning sustainability, resilience, and human-centricity as its core pillars. These elements are further explored in the following subsections.

### 2.2. Resilience-Based Maintenance Approach

Maintenance strategies have gradually evolved from reactive and scheduled maintenance to more proactive, data-driven approaches, such as predictive and condition-based maintenance. However, as industrial systems become increasingly complex, interconnected, and exposed to uncertain disruptions, traditional maintenance frameworks often prove insufficient in ensuring long-term system robustness and adaptability [53]. In response, resilience-based maintenance (RBM) emerges as a forward-looking strategy that enhances system capability to absorb, adapt, recover, and learn from disturbances [6].

The theoretical foundations of RBM are deeply rooted in resilience engineering [61,62,63], complex systems theory [64,65], and risk-informed asset management [66,67]. Here, resilience denotes a system’s ability to maintain or rapidly restore function amid disruptions [68,69]. RBM expands beyond fault prediction by integrating four core resilience capabilities [68,70,71]:

- Adaptability: The ability to adjust maintenance strategies and resource allocations dynamically in response to changing operational environments,
- Redundancy: The design and maintenance of alternative pathways or components (e.g., backup pumps, auxiliary control systems) to ensure continued function during partial failures,
- Learning: The use of historical and real-time data to continuously improve maintenance policies and failure response mechanisms,
- Recovery: The capacity to restore full system functionality rapidly following an adverse event.

**Figure 3:**
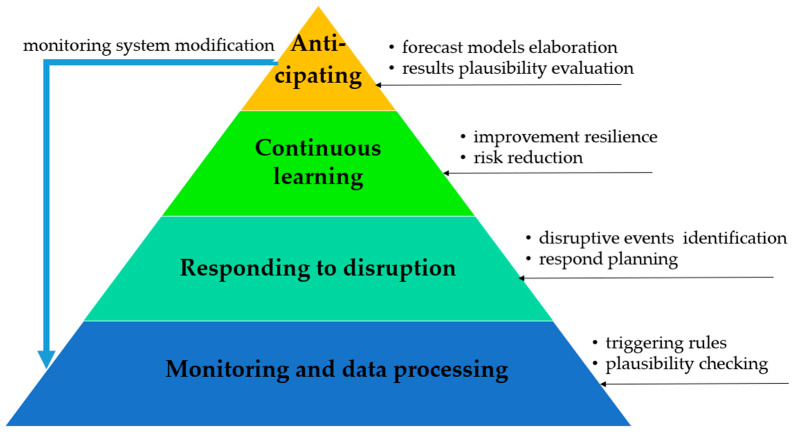
A layered architecture of RBM. Source: own contribution based on [6,74].

These capabilities, well-established in safety-critical domains [68,71,72], are now increasingly transferred to industrial maintenance, especially in high-reliability sectors [73]. Figure 3 illustrates the layered architecture of RBM.

RBM is gaining momentum as a research field. For example, Leng et al. [75] and Pawar et al. [76] map resilience strategies in manufacturing and maintenance, while [77] and [78] explore maintenance resilience under Industry 4.0 and 5.0 visions. These studies underscore the need to link resilience thinking with asset management and uncertainty response [6,7,79].

RBM distinguishes itself from reliability-centered maintenance and predictive maintenance (PdM) because of its explicit focus on system-level adaptability and human–machine interaction under dynamic and uncertain conditions [80,81]. While RCM relies primarily on structured failure mode analysis and expert knowledge to design preventive strategies [82,83,84], it often lacks the flexibility to respond to emerging disruptions. Predictive maintenance, on the other hand, utilizes sensor data and machine learning techniques to estimate the remaining useful life of components, offering significant benefits in condition monitoring. Yet, it focuses on specific assets or subsystems rather than the broader organizational context. RBM, in contrast, incorporates systemic foresight, learning feedback loops, and flexible resource reallocation, aligning with the principles of resilient socio-technical systems.

In recent years, the emergence of prescriptive maintenance (RxM) has further extended the capabilities of traditional predictive maintenance (PdM) [43]. While PdM focuses on estimating remaining useful life (RUL) and anticipating failures based on condition data, RxM introduces a decision-making layer that suggests or autonomously initiates optimal maintenance actions. This includes selecting corrective strategies, scheduling interventions, and even reconfiguring systems in real time based on risk, cost, and performance trade-offs. Prescriptive analytics leverages reinforcement learning, simulation-based optimization, and AI planning to enable autonomous maintenance decisions, often without human intervention. As such, RxM aligns closely with the adaptability and learning dimensions of resilience-based maintenance. However, unlike RBM, it typically lacks an explicit system-level perspective on socio-technical interactions, redundancy design, and organizational learning. Therefore, while RxM can be viewed as a technological enabler of RBM, it does not substitute the broader resilience paradigm. Integrating prescriptive maintenance within RBM frameworks offers a promising direction for developing intelligent, adaptive, and ethically grounded maintenance strategies [85,86].

**Figure 4:**
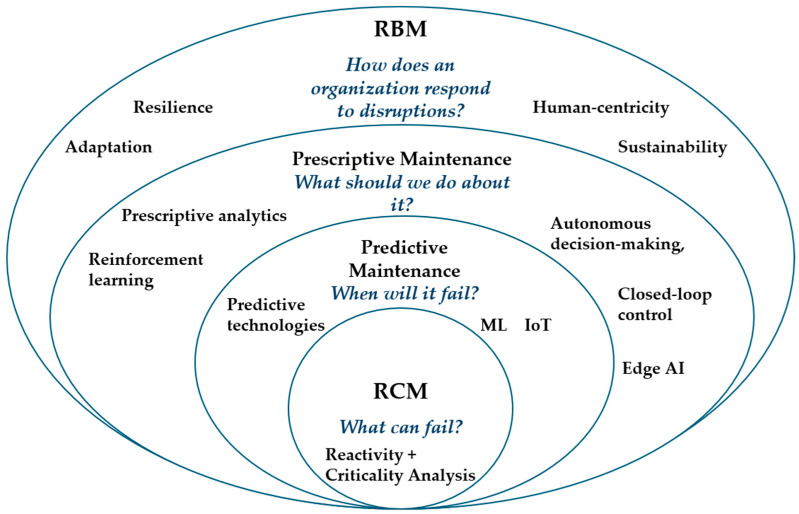
Conceptual boundaries and evolution from reliability-centred maintenance (RCM), predictive maintenance, and prescriptive maintenance (RxM) towards resilience-based maintenance (RBM).

To better understand the positioning of RBM in the landscape of maintenance strategies, it is essential to compare it with two established approaches, reliability-centered maintenance (RCM), predictive maintenance (PdM), and prescriptive maintenance (RxM) in a tabular form (Table 2). To clarify the conceptual evolution and scope of resilience-based maintenance (RBM), a concentric model has been introduced (Figure 4). This schematic visualizes how RBM builds upon the foundations of reliability-centred maintenance (RCM), predictive maintenance (PdM), and prescriptive maintenance (RxM), extending their focus beyond equipment-level reliability and prediction. RBM addresses systemic responses to disruptions, embedding resilience, flexibility, and sustainability into maintenance strategies. It also highlights the shift toward human-centric and ethically aligned asset management in Industry 5.0 contexts.

Despite rising interest, RBM remains fragmented in application. Existing literature often addresses resilience dimensions in isolation, e.g., AI for adaptability or learning, without integrating them into a unified operational framework. Moreover, few studies examine how RBM can be embedded within human-centric and sustainability-oriented strategies characteristic of Industry 5.0.

This article addresses these gaps by conducting a systematic literature review (SLR) on RBM and its intersection with AI, sustainability, and human-centricity in industrial maintenance. By synthesizing theoretical and practical developments, it proposes a structured framework to support adaptive and resilient maintenance in Industry 5.0 environments.

In summary, RBM offers a paradigm shift, from reactive interventions to proactive resilience orchestration, ensuring not only technical reliability but also long-term sustainability and human-centered design. This section lays the conceptual foundation for the subsequent classification and analysis of AI-based RBM strategies.

### 2.3. Sustainable Maintenance Approach

The concept of sustainability has become an indispensable element in transforming maintenance strategies, particularly within the Maintenance 5.0 paradigm [1,15]. As industries strive to meet environmental responsibility requirements, economic viability, and social equity, maintenance is no longer viewed solely as a technical function but as a strategic enabler of sustainable value creation. Sustainable maintenance refers to systematically integrating environmental, economic, and social criteria into maintenance planning, execution, and evaluation, ensuring that maintenance contributes to industrial systems’ long-term resilience and ethical operation [33,87].

The importance of sustainable maintenance has been addressed in several previous literature reviews, which provide a foundation for its conceptualization, challenges, and implementation. For instance, Campos and Simon [9] systematically examined how sustainability concepts are embedded into maintenance strategies in the context of sustainable manufacturing. Saihi et al. [33] conducted a modeling-based systematic review that identifies trade-offs and gaps in sustainable maintenance approaches. Zhao et al. [10] reviewed sustainable strategies for single and multicomponent equipment, while Hallioui et al. [11] analyzed total productive maintenance (TPM) in light of sustainability objectives. Vasić et al. [12] conducted an umbrella review that outlines critical criteria for adopting sustainable maintenance practices.

Additionally, several conceptual and application-oriented studies have contributed to the evolution of sustainable maintenance thinking. Durán and Durán [88] proposed a prioritization model for physical assets to align maintenance with sustainability goals in manufacturing. Kayan [89] introduced the notion of “green maintenance” from a conservation planning perspective, emphasizing material reuse and minimal environmental impact in repair processes. Olugu et al. [90] developed an integrated fuzzy Delphi–TOPSIS framework to identify sustainability indicators for maintenance in the oil and gas sector. Singh and Gupta [91] applied ISM–fuzzy MICMAC and TOPSIS to model interrelations among sustainable maintenance factors.

Recent research has also focused on the use of emerging digital tools to support sustainable maintenance. Rojek et al. [92] explored how digital twins can be applied across the product lifecycle to enhance maintenance planning and sustainability. Wu et al. [93] proposed a real-time, condition-based sustainable maintenance strategy using energy and performance data for milling processes. Patra and Dinesh Kumar [94] discussed opportunistic and delayed maintenance as tactical methods to optimize resource use and minimize disruptions, especially in constrained environments. Suresh and Dharunanand [95] identified key influencing factors for sustainable maintenance in manufacturing industries, including top management support, workforce competence, and regulatory pressure.

These contributions collectively demonstrate the broadening scope of sustainable maintenance, from environmental impact reduction to strategic integration of sustainability principles into decision-making, asset prioritization, and digitalization. However, they also reveal persisting challenges in harmonizing frameworks, metrics, and implementation tools across sectors and system scales.

In addition, recent surveys on sustainable maintenance problems are presented, e.g., in [31,32,33,35]. These prior works highlight the diversity of approaches and indicate that sustainable maintenance is not a single strategy but a multidimensional paradigm. Key issues include six main research areas investigated: green maintenance circular economy approach, energy efficiency, human-centered approach, life cycle assessment (LCA) principles implementation, and Industry 4.0 technologies. Additionally, regulatory and policy compliance ensures maintenance aligns with environmental regulations and corporate sustainability goals [96].

Among the emerging paradigms in sustainable maintenance, energy-based maintenance (EBM) has gained increasing scholarly attention as an integrative strategy linking maintenance practices with energy efficiency and sustainability goals. Unlike traditional predictive maintenance applied in energy systems [97,98], EBM explicitly focuses on energy consumption patterns as diagnostic indicators and bases maintenance decisions on energy-related metrics, such as power anomalies, energy efficiency trends, or energy-based availability of assets [99,100,101,102,103,104].

EBM is typically implemented through continuous monitoring of energy consumption, supported by smart metering infrastructure and advanced analytics. It enables the early detection of inefficiencies or degradation by correlating energy deviations with equipment health. This not only enhances equipment performance and reduces operational costs but also contributes to broader decarbonization and cleaner production objectives [105,106,107,108,109,110].

Recent studies have further advanced the EBM paradigm. For example, Orošnjak et al. [105,106,109] propose EBM as a step beyond predictive maintenance in achieving functional productiveness and cleaner production. Holmer et al. [104] introduce energy-based survival models for remaining useful life estimation, while Hoang et al. [102] apply EBM for decision support in manufacturing platforms. Moreover, researchers such as Erguido et al. [101] and Jiang et al. [103] integrate energy-efficiency considerations directly into opportunistic or condition-based maintenance policies. The strategic relevance of EBM has also been emphasized in sectors like wind farms [101], hydraulic machinery [106], PV plants [100], and conveyor systems [103].

As such, EBM reflects a paradigm shift from reliability-centric to energy-centric maintenance frameworks, combining operational performance with environmental responsibility. It aligns closely with the principles of Industry 5.0 by integrating AI-driven diagnostics, energy-aware modeling, and sustainability-centered decision-making.

**Figure 5:**
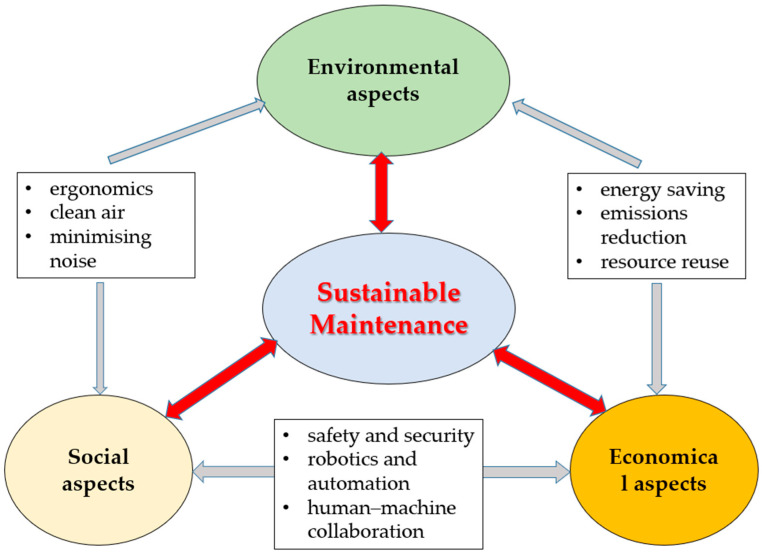
Conceptual model of sustainable maintenance integrating environmental, economic, and social dimensions within Industry 5.0. Source: own contribution based on [87,96,112].

Figure 5 illustrates the conceptual framework of sustainable maintenance, structured around the three core pillars of the triple bottom line (TBL) [111]: environmental, economic, and social sustainability. The model highlights how maintenance practices can simultaneously contribute to ecological efficiency, economic performance, and social responsibility. In addition, Table 3 introduces the key indicators of sustainable maintenance under the TBL Framework.

From an environmental standpoint, sustainable maintenance emphasizes minimizing ecological footprints through energy-efficient processes, life cycle-oriented spare part management, reduced material consumption, and the prevention of environmentally harmful failures. This includes the application of predictive and prescriptive analytics to optimize asset life cycles, using biodegradable lubricants or recyclable components, and the implementation of condition-based interventions that reduce unnecessary resource use [114]. Additionally, integrating digital technologies allows organizations to track environmental impacts in real time and align maintenance operations with broader sustainability reporting frameworks [32].

Economically, sustainable maintenance aims to reduce the total cost of ownership (TCO) by extending asset life, preventing costly breakdowns, and optimizing resource allocation [115]. Tools such as life cycle costing (LCC), risk-based maintenance, and AI-driven decision support systems contribute to achieving cost-efficient and performance-driven maintenance strategies. These tools also support better inventory management, spare parts logistics, and service contract optimization, directly linking maintenance actions with financial performance and asset management efficiency [116,117,118,119].

The social dimension of sustainable maintenance encompasses human well-being, ethical working conditions, and competence development. As outlined by the principles of Industry 5.0, sustainable maintenance recognizes the role of maintenance personnel as executors of tasks and critical knowledge holders and co-creators of intelligent systems. This involves promoting safety, ergonomics, transparency in decision-making, inclusion of workers in the design of smart maintenance tools, and lifelong learning opportunities. Worker-centric approaches, such as integrating augmented reality (AR) for task assistance or collaborative robots (cobots) in physically demanding tasks, also reduce physical and cognitive strain while enhancing job satisfaction and retention [120,121].

To better understand the distinguishing characteristics and underlying paradigms of evolving maintenance approaches, a comparative overview is presented in Table 4. This table contrasts the traditional, smart (Industry 4.0-based), and sustainable (Industry 5.0-aligned) maintenance models across key dimensions such as strategic focus, decision-making logic, enabling technologies, environmental and social considerations, and the role of human operators. By synthesizing the evolution from cost- and availability-driven strategies to those embedding broader values of sustainability and resilience, this comparative framework highlights how maintenance practices are progressively aligning with the principles of long-term value creation, ethical responsibility, and adaptive capability. The table serves as a conceptual bridge connecting technological transformation with the expanding expectations of industrial maintenance in the context of digitalization and socio-environmental responsibility.

Despite the growing interest in sustainable maintenance, recent literature highlights several challenges, including the lack of standardized indicators, fragmented data on environmental and social impacts, and limited integration of sustainability metrics in current maintenance management systems [111]. Multi-criteria decision-making (MCDM) methods (e.g., [112]), fuzzy logic-based evaluation models (e.g., [122]), and hybrid sustainability frameworks (e.g., [8]) are being proposed to overcome these barriers. These methods allow for aggregating qualitative and quantitative sustainability indicators and help organizations balance competing objectives across the triple bottom line (TBL).

In summary, sustainable maintenance is a multidimensional concept extending traditional maintenance boundaries toward broader sustainability goals. It operates at the intersection of ecological responsibility, economic efficiency, and social equity, thus aligning maintenance operations with the principles of Industry 5.0. The use of earlier systematic reviews in this field confirms the need for more integrative frameworks that connect sustainability, resilience, and AI-enabled maintenance management. In the following sections, the interconnections between sustainable maintenance, resilience engineering, and human-centric approaches are further examined, particularly in the context of AI-enabled decision-making and system adaptability. The interconnections between sustainable maintenance, resilience engineering, and human-centric approaches are further examined in subsequent sections, particularly in the context of AI-enabled decision-making and system adaptability.

### 2.4. Human-Centric Maintenance

As industrial systems evolve toward Industry 5.0, the human dimension becomes central to the design, implementation, and evaluation of advanced maintenance strategies [41]. Human-centric maintenance emphasizes the well-being, competence, and ethical inclusion of human operators in technologically advanced environments [20,123]. It acknowledges that while digital tools—such as AI, IoT, and cyber-physical systems—enhance decision-making and efficiency, they must support rather than replace human agency [124].

Moreover, socio-technical factors such as trust, explainability, and perceived control are critical for the successful implementation of AI-enabled resilience-based maintenance (RBM) strategies [125].

**Figure 6:**
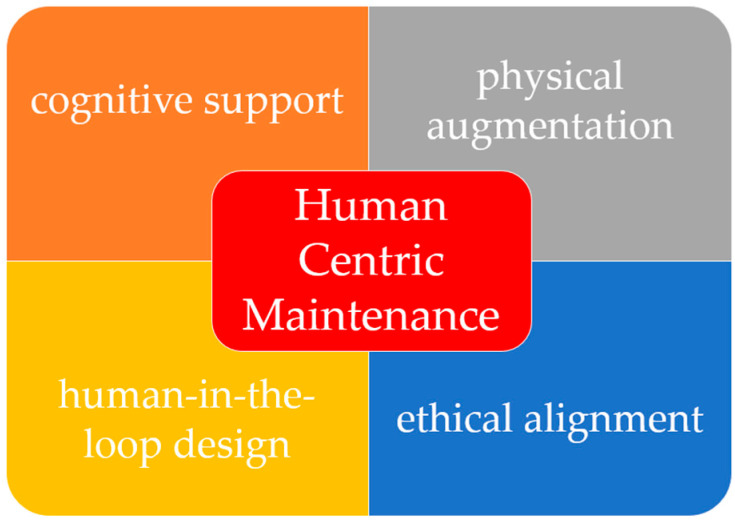
Key Dimensions of human-centric maintenance. Source: own contribution based on: [20,126,127].

Figure 6 illustrates the key dimensions of human-centric maintenance, highlighting the interaction between cognitive support, physical augmentation, human-in-the-loop (HITL) design, and ethical alignment. This conceptualization places the operator at the core of intelligent maintenance systems, emphasizing transparency, adaptability, and shared control. To illustrate these dimensions in practice, Table 5 maps selected technologies—such as AR, VR, and AI decision tools—to their human-centered features, including ergonomic support, transparency, and cognitive assistance.

Human-centric enablers often overlap with those used in RBM, such as AI, digital twins, machine learning, and knowledge graphs [4,14,26,29]. These technologies facilitate human–machine collaboration, maintain situational awareness, and support shared decision-making.

Recent studies have begun to explore these AI-driven capabilities within the RBM paradigm. The work of Kaewunruen et al. [129] highlights the potential of digital twins in enhancing resilience in railway maintenance, while studies by Ejjami & Khaoula [130] and Wiese [131] examine how AI-driven predictive maintenance can be used for improving the resilience of critical infrastructure and manufacturing systems. Additionally, the potential of AI in enhancing resilience across multiple domains is developed in [132].

This approach is closely linked to the Operator 4.0 concept, introduced by Romero et al. [133], which describes a set of human roles augmented by smart technologies in the context of smart factories. Within maintenance, Operator 4.0 may act as a super-strength operator (supported by exoskeletons), virtual operator (interacting through AR/VR), collaborative operator (working alongside robots or cobots), or analytical operator (supported by AI-based decision tools). These roles expand human capabilities while preserving the need for meaningful human participation, especially in decision-critical maintenance tasks.

A key principle of human-centric maintenance is human-in-the-loop (HITL) design. This involves embedding human operators into feedback loops within AI and automated maintenance systems, ensuring that human judgment can override, refine, or contextualize algorithmic decisions [134]. HITL is crucial for scenarios involving safety, ethical ambiguity, or uncertainty, where rigid automation might fall short. It also supports explainability, allowing users to understand how maintenance recommendations are generated and to trust AI systems accordingly [125,135]. The implementation of humans-in-the-loop in maintenance contexts is critically analyzed in works such as [136] and, more broadly, in production automation in [137].

Recent systematic literature reviews have highlighted the growing importance of this paradigm. For example, in [125], the authors focus on integrating AI with humans to enhance sustainability and customization in manufacturing. Human-centered AI issues are also reviewed in [138,139]. Another review by Verma [127] focused on human-centric and sustainable industrial revolutions in relation to the Industry 5.0 concept. It emphasizes that Industry 5.0’s human-centricity is not merely about user interfaces but a shift toward organizational culture, governance models, and participatory design. Similarly, the authors in [128,140] review human-centric smart manufacturing, emphasizing that the sustainability of human-centered systems and collaboration in hybrid environments will pose major challenges in future industrial systems. In addition, Pizon et al. [141] focus on the challenges of human-centered manufacturing, classifying them into four groups: social, technical, safety-related, and legal and ethical dimensions.

Furthermore, human-centric maintenance prioritizes ergonomics, safety, and worker satisfaction [142]. It calls for both efficient but physically and cognitively adaptive systems, reducing fatigue, preventing injuries, and minimizing stress caused by complex digital interfaces. This includes intuitive HMIs (human–machine interfaces), AR-assisted diagnostics, and digital twins that help visualize machine states in user-friendly formats [143,144]. In addition to ergonomic and cognitive considerations, human-centric maintenance must also address the evolving standards of worker safety in increasingly automated and AI-driven environments. As maintenance systems become more complex and autonomous, ensuring safety requires compliance not only with traditional occupational health and safety standards (e.g., ISO 45001 [145], IEC 61508 [146]), but also with emerging guidelines on AI governance and ethics, such as the ISO/IEC 42001:2023 [147] standard for AI management systems and the EU AI Act [148]. These frameworks aim to support the safe, transparent, and accountable deployment of AI technologies in industrial settings. However, current documentation and regulatory texts may be subject to ambiguous interpretations, especially in hybrid human–machine contexts [149]. This complexity poses a challenge for practitioners seeking to ensure compliance while adopting cutting-edge AI-based tools in maintenance.

As emphasized by Khurram et al. [149], aligning Maintenance 4.0 practices with human-centric values and safety standards requires an integrative approach that combines predictive technologies with explainability, fail-safes, and risk-aware system design. This includes embedding worker-safety protocols in autonomous operations and ensuring that decision-support systems enhance, rather than obscure, human oversight. As such, human-centric maintenance is inseparable from the broader regulatory ecosystem shaping the industrial application of AI. Recent reviews that summarize a human-centric approach in Industry 5.0 are, among others, [150,151].

Table 6 presents a comparative view of human-centric attributes across maintenance generations from Maintenance 1.0 to Maintenance 5.0, aiming to contextualize the evolving role of the human within maintenance practices. This idealized, strategic model illustrates the shift from reactive, physically demanding maintenance toward ethically integrated, cyber-physical systems that protect and empower workers within intelligent infrastructures. It is essential to recognize that the model is normative in nature—it does not depict the current state of industrial practice but rather outlines a desired trajectory. As such, it serves a forward-looking, strategic function: to support organizations in identifying development gaps, planning transformation pathways, and assessing their readiness to move toward higher levels of maturity in maintenance. In practice, however, progress toward Maintenance 5.0 is rarely linear and is often obstructed by organizational resistance, skill mismatches, trust issues surrounding AI, and high implementation costs. These challenges, captured in the “Transition Barriers” row, are consistent with broader findings in current literature and include not only socio-technical resistance, but also the regulatory uncertainty associated with interpreting and applying evolving AI and safety standards in practice. For instance, the European Commission [2] emphasizes that the success of Industry 5.0 hinges not only on technological readiness but also on managing social and ethical transitions, including workforce acceptance and the responsible deployment of AI technologies. Similarly, McKinsey & Company [152] highlights that many firms face deep-seated cultural and operational obstacles that require comprehensive change management strategies. Empirical studies echo these concerns, identifying a lack of trust, digital skill shortages, and resistance to change as critical barriers to transformation [153]. By framing the model as an inspirational planning tool, rather than a descriptive snapshot, it becomes a valuable aid for aligning strategic vision with actionable steps toward more human-centric and sustainable maintenance systems.

In summary, human-centric maintenance provides the ethical and operational foundation for integrating AI into maintenance in a way that empowers people, enhances system adaptability, and supports the Industry 5.0 vision. It is inseparable from RBM and sustainability, forming a key pillar of Maintenance 5.0.

## 3. Review Methodology

### 3.1. Review Design and Protocol

This systematic literature review was conducted in accordance with the preferred reporting items for systematic reviews and meta-analyses (PRISMA 2020) guidelines [47,154], which provide a structured and transparent framework for reporting evidence-based reviews. Adopting this protocol ensures methodological rigor, transparency of reporting, and reproducibility of the search and selection processes, minimizing bias and improving the validity of findings.

The overarching aim of this review was to critically examine the evolution and current state of resilience-based maintenance (RBM) within the broader transition toward Maintenance 5.0. Particular attention was paid to integrating artificial intelligence (AI) technologies, human-centric and sustainable design principles, and their interplay with resilience engineering and maintenance strategy development. The review also identifies gaps, limitations, and future research directions by mapping existing studies to key theoretical domains and application areas.

This review does not limit the analysis to a particular sector, as the focus is on conceptual developments and methodological frameworks of RBM. Therefore, relevant studies from the infrastructure, manufacturing, and energy domains were included.

**Figure 7:**
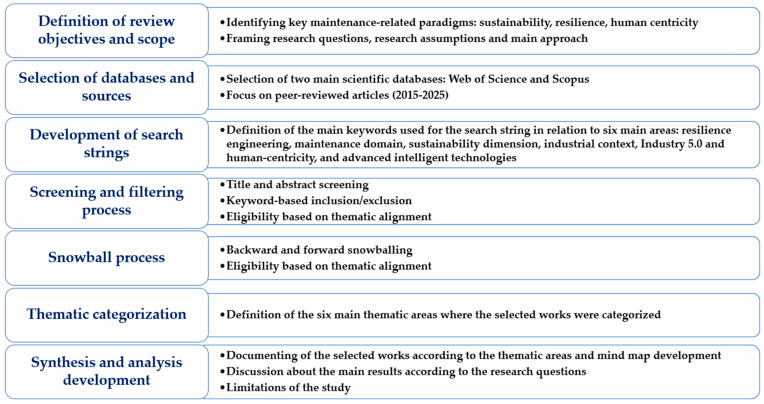
Research framework and methods/tools used for systematic literature review. Source: own contribution.

A structured review protocol was developed to guide the process in five main stages and was designed according to the principles given in [155,156,157,158]. These stages are illustrated in Figure 7, which presents the overall methodological framework used in this review:

- Definition of research objectives and questions—establishing the scope of the evaluation, including the conceptual focus on RBM and its relation to other maintenance paradigms under Industry 5.0.
- Search strategy development—formulating a comprehensive query string and selecting relevant databases (Scopus, Web of Science).
- Screening and eligibility assessment—applying inclusion and exclusion criteria, removing duplicates, and performing title/abstract and full-text screenings,
- Supplementary search—using snowballing techniques (both backward and forward citation tracking) to enhance literature coverage.
- Data extraction, synthesis, and classification—analyzing and categorizing the final set of articles by themes, methods, application domains, and contributions.

**Figure 8:**
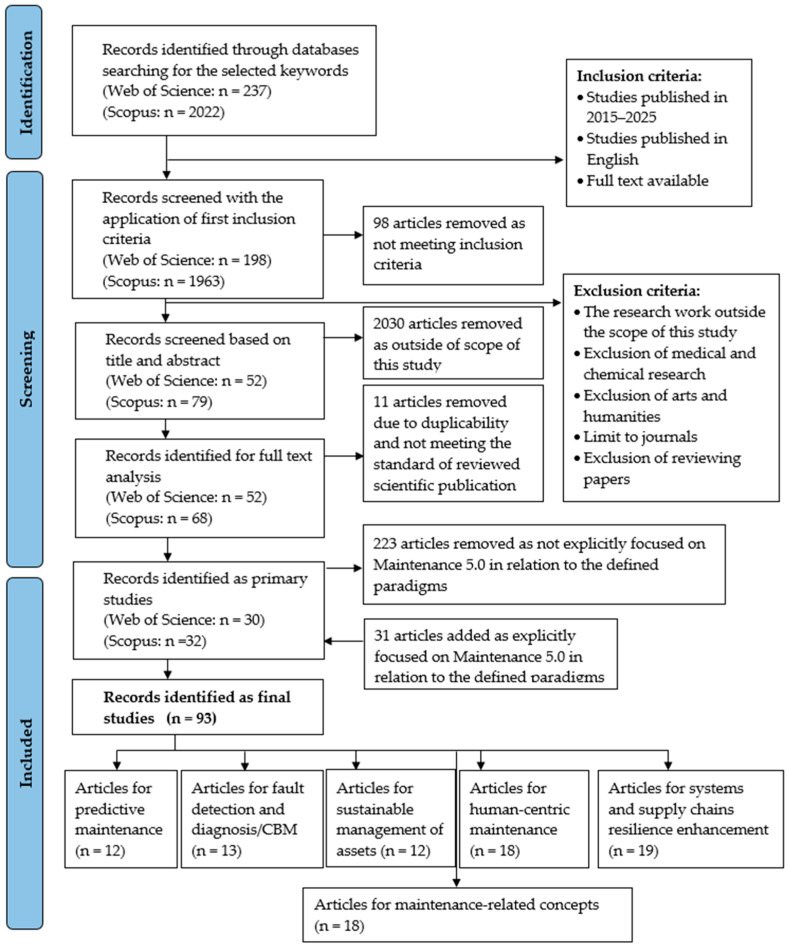
PRISMA-based flowchart of systematically selecting relevant studies in the analyzed research area. Source: own contribution based on [47].

This five-step workflow enabled a focused and replicable identification of scholarly contributions relevant to RBM, emphasizing theoretical advancements and practical implementations. In subsequent sections, the detailed execution of each stage is described, and the search results are summarized using a PRISMA flow diagram (Figure 8). The PRISMA checklist is given in Table S1 in the Supplementary Material.

### 3.2. Identification—Search Strategy

The identification stage of this systematic literature review focused on capturing a broad yet thematically coherent set of publications relevant to the evolving field of resilience-based maintenance (RBM) within the context of Industry 5.0. The search strategy was designed to reflect the interdisciplinary nature of the subject, combining technical, organizational, and human-centric perspectives.

To ensure methodological transparency and relevance, the search process was carried out in two leading academic databases: Web of Science (WoS) and Scopus. These databases were selected due to their comprehensive indexing of high-impact, peer-reviewed publications across engineering, manufacturing, and management domains. The searches were conducted during the period from 1 June 2025 to 15 June 2025.

The selection of keywords and Boolean logic operators was based on an initial scoping review of the literature, during which representative papers from different disciplines (maintenance engineering, resilience theory, human factors, AI applications) were reviewed to identify recurring terms, concepts, and taxonomy. This preliminary phase ensured that the final query captured the diversity of terminologies used across academic communities while aligning with the conceptual focus of this study.

The final search string was organized around six major thematic blocks, using the ALL fields search mode:

- Resilience engineering: Resilience OR robustness OR adaptability OR recoverability,
- Maintenance domain: Maintenance OR upkeep OR repair OR service OR fault OR failure OR diagnostics OR diagnosis OR prognosis OR inspection OR monitoring,
- Sustainability dimension: Sustainable OR eco-friendly OR green OR environmental-friendly OR circular OR energy-efficient,
- Industrial context: Industrial systems OR manufacturing OR production OR operations OR industrial processes,
- Industry 5.0 and human-centricity: Industry 5.0 OR human-centric OR human-centered OR user-centered OR people-oriented OR social OR human factor OR human–machine interaction OR human-in-the-loop OR anthropocentric OR ergonomics,
- Advanced intelligent technologies: Artificial intelligence OR AI OR digital twin OR smart system OR intelligent system OR machine learning OR cyber-physical system OR IoT OR big data OR cloud computing OR edge computing OR augmented reality OR AR OR virtual reality OR VR OR blockchain.

Each term group was connected using the Boolean AND operator to ensure conceptual coherence across resilience, maintenance, sustainability, human-centric design, and smart technologies.

In Web of Science, this initial query returned 237 records. Applying a publication date filter (2015–2025) narrowed this number to 198. Review papers and non-original research documents were then excluded, yielding 174 articles. Subsequently, publications falling outside the technical and engineering scope (particularly in medicine and chemistry) were eliminated, leaving 174 (unchanged due to query refinement). A detailed relevance screening based on titles, abstracts, and keywords led to the selection of 52 articles for full-text review.

In Scopus, the initial search retrieved 2022 records, spanning publication years from 1963 to 2025. These were first limited to the 2015–2025 range and filtered by document type, excluding review papers, book chapters, and conference papers. This reduced the dataset to 827 documents. Further exclusions targeted irrelevant subject areas such as medicine, humanities, and the arts, leaving 762 articles. Additional filters were applied to ensure consistency: only English-language documents (755) published in peer-reviewed journals (706) were retained. Following the same screening process based on titles, abstracts, and keywords, 79 articles were deemed relevant for full-text analysis. After removing 11 duplicates across both databases, a final pool of 120 unique publications was established for in-depth evaluation.

### 3.3. Screening—Eligibility Criteria

The screening process consisted of two phases: *(a)* preliminary filtering based on metadata and *(b)* content-based eligibility assessment, conducted to ensure methodological rigor and thematic consistency.

In the preliminary phase, exclusion criteria were applied to eliminate documents that did not meet basic methodological or topical thresholds. Specifically, papers that were (1) not published in peer-reviewed journals, (2) not written in English, or (3) classified as review articles, conference papers, book chapters, or editorials were excluded. Subject area filters were also employed to discard articles unrelated to engineering, manufacturing, operations, or maintenance, most notably those focused on medical, biological, or chemical applications. To increase transparency and facilitate replicability, the inclusion and exclusion criteria applied during the screening process are summarized in Table 7.

In the second phase, all remaining articles were evaluated for eligibility based on thematic relevance, using a set of predefined inclusion criteria. Publications were retained if they addressed one or more of the following topics:

- Resilience, robustness, adaptability, or recovery in the context of industrial maintenance,
- Integration of sustainable or circular principles into maintenance strategies,
- Human-centric approaches (e.g., human-in-the-loop, Operator 4.0, ergonomics) in industrial systems,
- Application of smart or intelligent technologies such as AI, digital twins, IoT, or cyber-physical systems in maintenance practices.

This phase relied on a detailed manual review of titles, abstracts, and keywords. The process was guided by a protocol aimed at maximizing both precision (removal of non-relevant articles) and recall (preservation of diverse but related studies).

Ultimately, 120 articles (after removing duplications) met all eligibility criteria and formed the basis for the subsequent synthesis and classification phases of the review.

### 3.4. Inclusion—Full-Text Review and Selection

The final step of the selection process involved a full-text review of the remaining articles, ensuring that each included study met the quality standards and contributed substantially to the review’s objectives.

A total of 120 records (68 from Scopus and 52 from Web of Science) were considered for full-text evaluation. After detailed reading and critical appraisal, approximately 62 publications were retained for qualitative synthesis. These studies provided a comprehensive representation of current trends, challenges, and future directions in resilience-based maintenance and related approaches under digital, sustainable, and human-centered paradigms.

The full-text review followed these detailed criteria:

- Relevance: Articles had to present explicit models, frameworks, case studies, or methodologies related to RBM, predictive maintenance, or sustainability in industrial systems.
- Methodological soundness: Publications were assessed for clarity of objectives, rigor in methodology, and robustness of results.
- Contribution to knowledge: Only articles that offered conceptual advances, empirical findings, or practical insights were included.

The evaluation was performed by two independent reviewers with expertise in the fields of maintenance engineering and system resilience. A collaborative spreadsheet was used to track decisions and notes, and any discrepancies were resolved through dialogue and reference to the study’s relevance criteria.

This multi-stage inclusion process ensured a rigorous and transparent selection of high-quality sources that formed the foundation for the subsequent synthesis and discussion. Additionally, although PRISMA guidelines recommend measuring interrater agreement to assess the consistency between reviewers, no formal interrater agreement score (e.g., Cohen’s kappa) was calculated in this review. The screening process was conducted jointly by two authors, who discussed and resolved inclusion decisions collaboratively. This approach, while transparent, may introduce potential subjectivity in the eligibility assessment.

### 3.5. Snowball Process—Final Selection

To complement the database-driven search strategy and to ensure the inclusion of relevant but potentially overlooked literature, the snowballing technique was employed as an additional retrieval method. This approach followed guidelines proposed by Wohlin [48] for systematic literature reviews in software and engineering domains, and it was executed in both backward and forward directions.

Backward snowballing involved screening the reference lists of all publications that were shortlisted after the full-text review stage (Section 3.4). Each cited work was evaluated for potential relevance to resilience-based maintenance (RBM), predictive or sustainable maintenance approaches, and applications within industrial contexts aligned with Industry 4.0 and 5.0 paradigms. If a referenced article met the previously established inclusion criteria (e.g., relevance, peer-reviewed status, methodological rigor), it was retrieved and subjected to the same screening and inclusion process.

Forward snowballing was conducted using Scopus citation tracking tools, which allowed for the identification of publications that had cited the initially selected core articles. This step was particularly useful for identifying emerging studies published after the core literature, including high-impact conceptual or empirical contributions to the development of RBM, AI-enhanced maintenance strategies, and the integration of human-centric or sustainable principles.

Through snowballing, an additional set of relevant articles was retrieved and considered for inclusion. While the majority of relevant literature was captured through the structured search queries in Scopus and WoS, snowballing added both depth and breadth to the review, uncovering niche studies and older seminal works frequently cited in contemporary research. Finally, 15 articles were selected for final review.

### 3.6. Documenting the SLR Study

This stage corresponds to steps 7, 8, and 9 of the systematic literature review (SLR) process and focuses on the documentation, analysis, and synthesis of the selected literature. Following the selection of eligible papers, a bibliometric analysis was conducted to gain deeper insight into the intellectual structure and thematic distribution of the field. Bibliometric methods, widely used in scientometric research, apply statistical and mathematical techniques to assess scientific activity and identify trends, influential contributors, institutional affiliations, and geographic distribution of research outputs.

In this study, bibliometric mapping and analysis were performed using VOSviewer (v. 1.6.18) [159] and Microsoft Excel (Professional Plus 2019). VOSviewer enables the construction and visualization of bibliometric networks, particularly focusing on co-authorship, keyword co-occurrence, citation relationships, and country-level collaboration [160]. These analyses helped identify dominant research clusters, recurring keywords (e.g., “resilience,” “predictive maintenance,” “Industry 5.0”), and key contributing countries and institutions. The keyword co-occurrence map and author collaboration network generated in VOSviewer are presented and discussed in Section 4.

The selected corpus of articles was further organized and managed using Mendeley reference management software [161], which facilitated both qualitative review and traceability. Each entry was tagged with metadata such as publication year, journal or conference source, research type (empirical, conceptual, review), and relevance to the key research themes outlined in Section 2.

Step 8 of the SLR process involved the content-based synthesis of findings in alignment with the review’s objectives and guiding questions. The synthesis process examined how the themes of resilience, AI integration, and sustainable maintenance were conceptualized and operationalized across various studies. These insights inform the thematic analysis presented in Section 4 and Section 5.

Finally, step 9 involved the reflection on limitations and the identification of future research directions. Limitations included a potential exclusion of relevant works not indexed in the selected databases or published in non-English languages, as well as the inherent subjectivity in interpreting conceptual overlap across domains such as resilience and predictive maintenance. These limitations and the derived future research avenues are critically discussed in Section 6.

## 4. Systematic Literature Review Results

This section includes the results of the conducted systematic review according to the defined research methodology (Figure 7). As a result, in the next subsections, bibliometric analysis and content-based analysis are presented.

### 4.1. Bibliometric Analysis

This section presents the quantitative and network-based characteristics of the selected literature to provide an overview of the scientific landscape in the field of resilience-based maintenance (RBM) within the broader context of Industry 5.0, which integrates sustainability, human-centricity, and intelligent technologies.

The bibliometric analysis was performed on a consolidated dataset of 93 unique articles, retrieved from the Scopus and Web of Science (WoS) databases, including snowball analysis and covering the publication period 2015–2025. The bibliographic data were exported in compatible formats and processed using VOSviewer (version 1.6.18), a specialized tool for constructing and visualizing bibliometric maps. The largest number of articles (19 papers) was in two of the defined areas: systems and supply chains resilience enhancement, and maintenance-related aspects. The number of analyzed publications in the other areas is as follows: human-centric maintenance—18 papers; fault detection and diagnostics/CBM—13 papers; predictive maintenance in Industry 4.0—12 publications; and sustainable management of assets and environmental impact—12 publications.

The analysis of the selected articles focused on the following dimensions:

- Publication dynamics and source distribution—to identify temporal patterns and the increasing attention toward RBM-related topics, as well as to examine the scientific outlets (journals and conference proceedings) in which these studies are most frequently published, thereby revealing the disciplinary focus and visibility of the field.
- Country-level collaboration—to examine the geographic spread and international cooperation in RBM research.
- Co-authorship networks—to explore collaboration patterns among authors and institutions.
- Co-occurrence of keywords—to identify thematic clusters, trends, and emerging research areas.

In particular, keyword co-occurrence analysis was used to visualize the conceptual structure of the field. The threshold was set to include terms appearing at least 1 time in the dataset, which resulted in a network of interconnected keywords grouped into several major clusters. These clusters reflect distinct yet interrelated themes such as Industry 4.0, Industry 5.0, resilience, predictive, and sustainability in industrial systems.

The network visualization maps generated using VOSviewer illustrate the relative importance of terms (node size), their co-occurrence strength (link thickness), and thematic proximity (color-coded clusters). The results provide an empirical foundation for interpreting the knowledge structure of RBM and its positioning within Maintenance 5.0 discourse.

**Figure 9:**
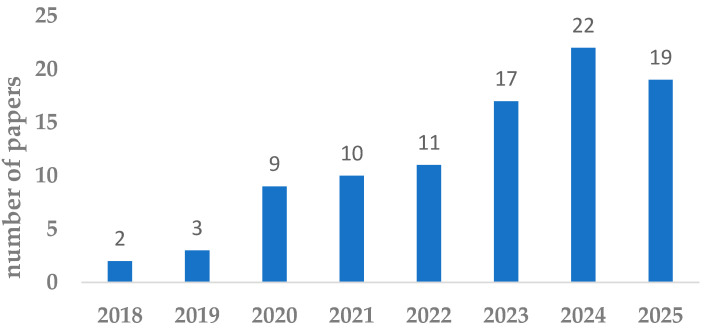
Distribution of publications by year.

**Figure 10:**
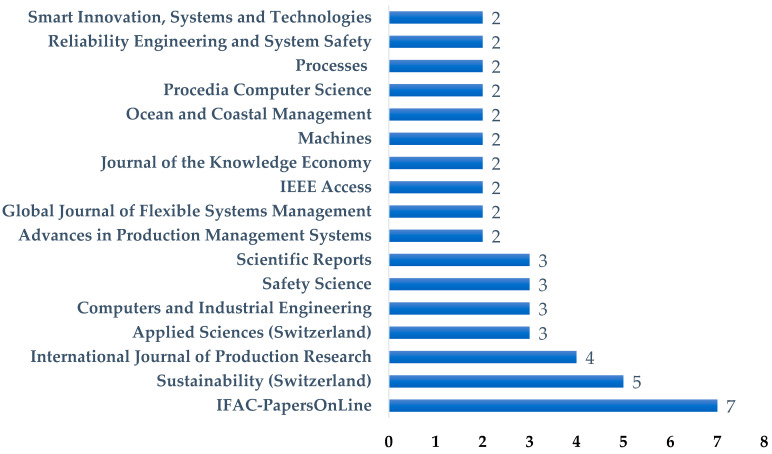
Number of publications with journal sources (for journals with at least two published articles out of the 93 articles analyzed).

**Figure 11:**
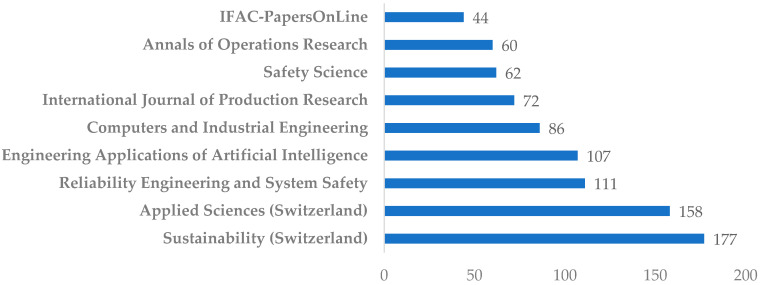
The number of citations for the analyzed journals (top-cited journals from the dataset).

**Figure 12:**
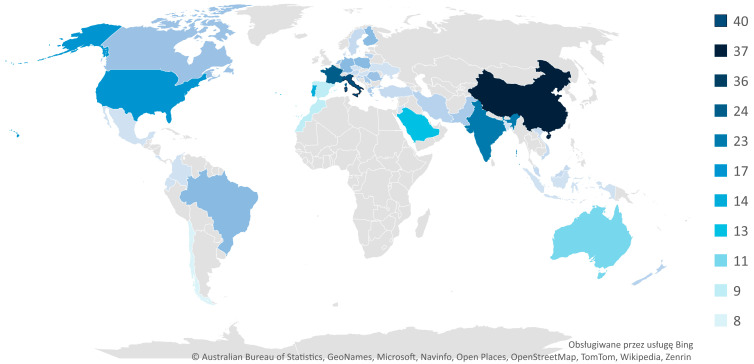
The number of papers by the location where the investigated study took place.

The outputs of the conducted analysis are presented in Figure 9, Figure 10, Figure 11 and Figure 12.

First, the publication dynamics were investigated. In the conducted review, the authors selected 93 publications that were published between 2015 and 2025. Figure 9 illustrates the distribution of the publications according to their publication year. As we can see, the distribution of publications by year starts from 2018 and is slightly increasing year by year. This is connected with the fact that before 2018, the problem of Maintenance 4.0 in joint relation to resilience and sustainability had not been investigated at all. The first publications in this field were observed in 2018 and related to robustness in terms of resilience or sustainability. The highest number of publications falls within the period 2023–2025, with 58 publications accounting for 62% of all publications selected for analysis. On one hand, the observed trend is consistent with global research and innovation agendas, which have increasingly emphasized resilient, sustainable, and digital industrial systems. On the other hand, the formal introduction of the Industry 5.0 paradigm in 2021 by the European Commission has catalyzed a broader conceptual shift. Since then, greater attention has been directed toward integrating digitalization not only with resilience but also with sustainability and human-centricity.

This renewed focus has significantly influenced the maintenance domain, where traditional optimization-centered strategies are being re-evaluated in light of ethical, environmental, and social considerations. Consequently, scholarly contributions are now more likely to adopt interdisciplinary perspectives that combine artificial intelligence, circular economy principles, and human–machine collaboration, marking a paradigm shift toward Maintenance 5.0.

Moreover, the analyzed articles were published across 55 different journals, reflecting the wide-ranging and interdisciplinary nature of the research on resilience-based maintenance (RBM) within the broader framework of Industry 5.0. Figure 10 illustrates the distribution of journals that included at least two publications relevant to the topic.

In total, 48 articles from 17 journals met this criterion and were considered in the source distribution analysis. The dataset included both journal articles and peer-reviewed conference proceedings, with IFAC-PapersOnLine, a leading venue for automation and control research, being the most represented source.

Among the sources, *IFAC-PapersOnLine* emerged as the most frequent publication outlet, contributing seven articles to the selected corpus. The journal *Sustainability* (Switzerland) ranked second with five publications, followed by the *International Journal of Production Research* with four articles. Additionally, *Applied Sciences, Computers and Industrial Engineering, Safety Science,* and *Scientific Reports* each contributed three publications, indicating a consistent, albeit dispersed, interest across various fields.

This broad distribution of publication venues confirms the interdisciplinary character of the field, bridging areas such as industrial engineering, production management, artificial intelligence, safety science, and sustainability. The prominence of *IFAC-PapersOnLine* underlines the relevance of the topic within automation and control systems communities, while the presence of journals like *Sustainability* and *Scientific Reports* suggests an increasing emphasis on the integration of environmental and societal dimensions into maintenance research.

Such dispersion also implies that research on RBM is still emerging and expanding, not yet consolidated around a small number of flagship journals. This may offer opportunities for wider dissemination, but also indicates the need for more focused publication channels as the field matures.

To assess the scientific influence of the reviewed publications, citation metrics were calculated using Scopus citation counts. Among journals with at least three publications in the selected corpus, *Sustainability* and the *International Journal of Production Research* (IJPR) stood out in terms of citation performance.

For articles published in *Sustainability* that have received at least 10 citations, the average citation count was 21.3 citations per article, with a median of 19. In comparison, articles published in IJPR achieved an average of 26.8 citations, with a median of 23. These figures indicate that research on resilience-based maintenance (RBM), particularly when linked to sustainability and production systems, is gaining substantial visibility and scholarly traction. Figure 11 presents the most cited journals from the dataset. Number of citations per selected papers according to Scopus database (papers with minimum 1 citation) is presented in Appendix A.2, Table A2.

The citation performance reflects both the growing relevance of RBM in the context of Industry 5.0 and the role of these journals as preferred publication venues for interdisciplinary studies at the intersection of AI, resilience engineering, and maintenance strategy.

As part of the bibliometric analysis, the geographical origin of the selected publications was examined to understand the global distribution and intensity of research activity related to resilience-based maintenance (RBM) in the context of Industry 5.0. The results show a wide international interest in this emerging area, with contributions from 45 countries across six continents.

Europe is the most prominent region, accounting for approximately 54% of all analyzed publications (175 documents), followed by Asia with 24% (77 publications), and Africa with a notable 9% (28 publications). The remaining contributions are from North and South America (22 and 20 publications, respectively), and Australia/Oceania, which together represent a smaller but still relevant share (14 publications).

At the country level, the top contributors are Italy (40 publications), China (37), and the United Kingdom (36), followed by France (24) and India (23). The United States, often leading in digital innovation, appears with a moderate contribution of 17 publications. Other countries with visible activity include Portugal (14), Saudi Arabia (13), Australia (11), Spain (9), and Morocco (9). Notably, countries such as Germany, Poland, Finland, and Brazil each contributed seven articles, reflecting a balanced representation between highly industrialized nations and those undergoing digital transformation.

This distribution reflects the growing relevance of RBM not only in traditional centers of technological advancement but also in emerging economies and regions where industrial resilience and sustainable practices are becoming increasingly important. The presence of countries such as Morocco, Pakistan, and Romania demonstrates the expanding global engagement with Industry 5.0 principles and maintenance modernization.

From a regional perspective, Europe’s leadership in publication volume may be attributed to its strong policy emphasis on sustainable development, digitalization, and human-centric technologies, as reflected in EU-level initiatives supporting Industry 5.0 research. Asia’s significant share, with major contributions from China and India, indicates the region’s rapid technological adoption and increasing interest in resilience-based strategies across sectors. Africa’s representation, unusually high compared to other technical domains, may point to growing awareness of infrastructure sustainability and capacity-building in critical industries.

Figure 12 presents the distribution of RBM-related publications across the top contributing countries and their relative publication output.

**Figure 13:**
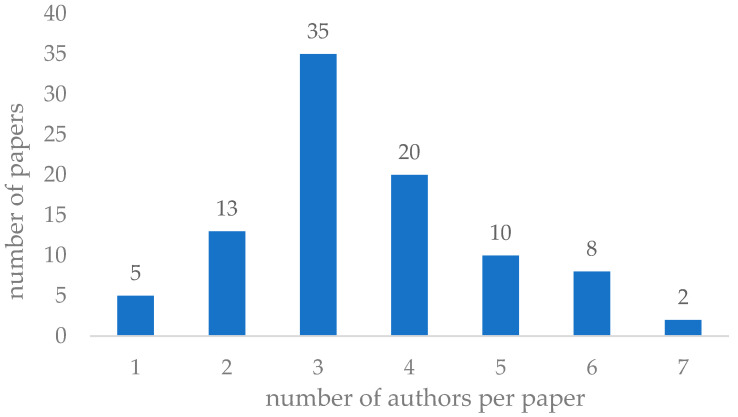
Distribution of publications per number of authors.

To complement the bibliometric exploration, co-authorship patterns were examined. In total, the dataset included 93 articles, co-authored by a diverse pool of researchers. The number of authors per article was analyzed to identify collaboration intensity. As shown in Figure 13, the most common authorship model includes three authors (35 articles), followed by four (20 articles) and two (13 articles). Notably, single-author contributions were relatively rare (5 articles), suggesting a high degree of collaborative research in the analyzed field. In addition, articles with larger teams (six or more authors) represented only 10.7% of the total sample (10 articles), indicating that although collaboration is prevalent, extensive multi-author projects are less frequent in the RBM and Maintenance 5.0 literature.

In the next step, a co-authorship network was constructed using VOSviewer to identify the most collaborative researchers within the dataset.

The top 15 authors by total link strength and number of joint publications were extracted. The analysis reveals that collaboration networks are predominantly localized within country-specific clusters (e.g., Italy, China, India), with emerging transnational links in European-funded or interdisciplinary studies.

**Figure 14:**
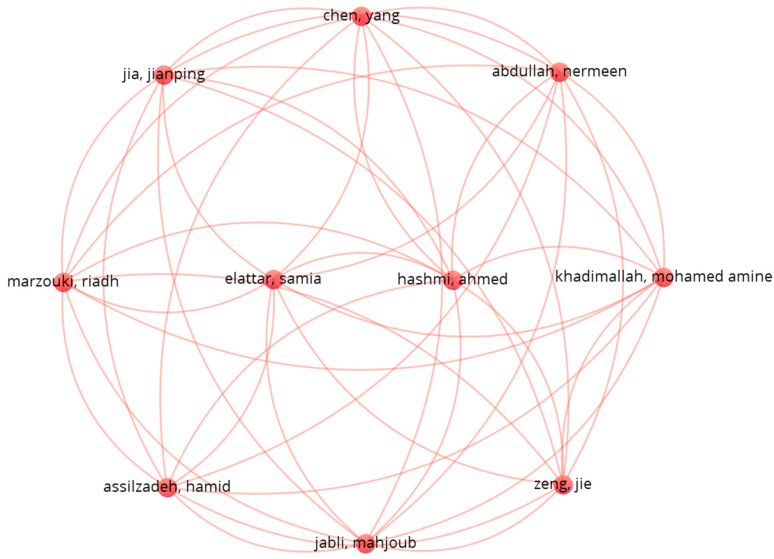
The largest set of connected items based on co-authorship links. Source: own development using VOSviewer software [159].

In order to supplement the conducted analysis, a co-occurrence of authors was investigated using VOSviewer software and Excel software. A total of 328 authors were identified from the selected papers. Figure 14 shows the results for the top 10 authors with the highest number of co-authorship connections. The authors presented in this cluster have the largest total link strength (9).

**Figure 15:**
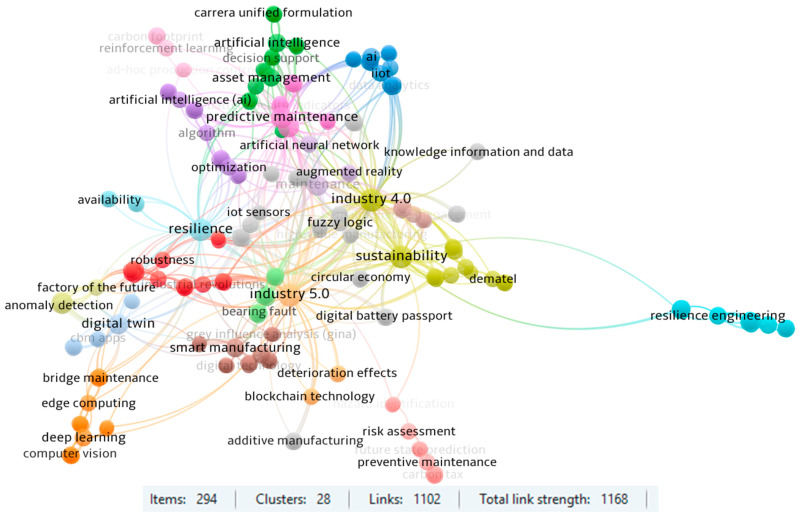
Mapping of the keywords that have occurred in the selected publications at least once. Source: own development using VOSviewer software [159].

The final part of the bibliometric investigation was dedicated to keyword co-occurrence analysis based on author-provided keywords, using VOSviewer software. In total, 294 unique keywords were identified across the analyzed publications, forming 28 distinct clusters. These clusters were connected through 1102 co-occurrence links with a total link strength of 1168, reflecting a dense and multidimensional thematic structure (Figure 15).

The most frequently used terms included Industry 5.0 (80 links), Industry 4.0 (75), resilience (72), sustainability (66), predictive maintenance (48), and machine learning (43). These keywords clearly reflect the prevailing focus areas within the field, indicating a strong alignment between human-centric innovation, advanced digital technologies, and proactive maintenance strategies.

The largest cluster (red one, 20 items) is centered around the role of human capital and cyber-physical integration in the context of Industry 5.0. It emphasizes the transformation of maintenance systems through technologies such as automation, collaborative robotics (cobots), and advanced cyber-physical systems, supporting human–machine cooperation. The second largest cluster (green one, 19 items) pertains to asset management supported by artificial intelligence, reflecting a growing emphasis on intelligent decision support systems in resilient maintenance frameworks. The third cluster (light blue, 18 items) encompasses terms related to the integration of AI, IoT, and data analytics into predictive maintenance, which form the foundation of many Industry 4.0-driven solutions.

Another important thematic area (yellow cluster, 18 items) highlights sustainability and environmental considerations, emphasizing issues such as circular economy, energy efficiency, and eco-friendly maintenance practices. This is complemented by a fifth cluster (magenta, 17 items) focused on optimization and decision-making, particularly in AI-enabled industrial systems. Equally significant is the sixth cluster (cyan, 17 items), which addresses resilience engineering and risk management, underlining the role of proactive strategies to enhance system reliability under uncertainty. The seventh major cluster (orange, 17 items) shifts attention toward human factors, highlighting topics such as ergonomics, deep learning, and computer vision, especially in the context of monitoring and supporting operator performance.

The remaining clusters, each comprising between three and sixteen keywords, reflect niche areas such as digital twins, maintenance optimization, reliability analysis, and the implementation of circular economy principles. Together, these findings present a comprehensive overview of how research in resilience-based and sustainable maintenance is structured around interconnected yet distinguishable thematic pillars, offering insights into the future directions of the field.

The presented bibliometric investigation enhances understanding of how RBM research has evolved and which directions are emerging, while also supporting the formulation of content-based insights discussed in Section 4.2.

### 4.2. Content-Based Analysis

As a result of the conducted research, following the methodology adopted (Figure 7), we focus on the content-based analysis.

**Figure 16:**
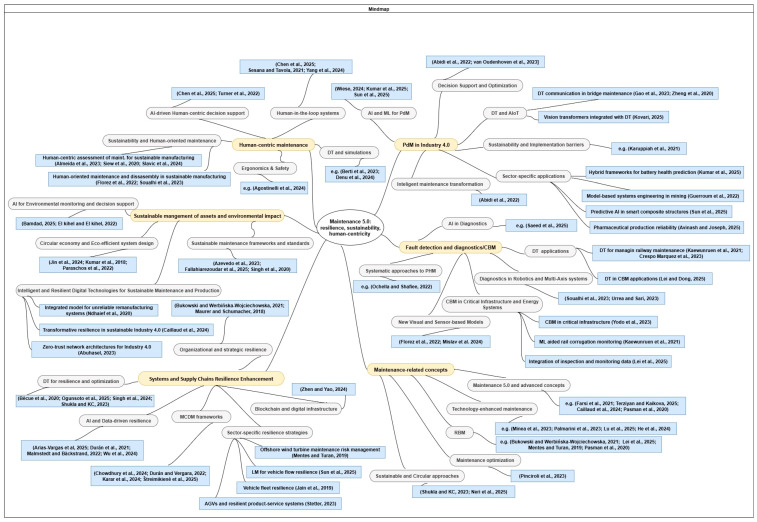
Mind map presenting the six major thematic areas of resilience-based maintenance (RBM) in the context of Industry 4.0 and 5.0, including key research topics and associated publications. Source: own contribution with the use of the draw.io app.

The identification of the main problems and issues raised in the context of Maintenance 5.0 was based on an extensive review of the available literature. As a result of the research carried out, six core research areas were defined, which have been most extensively developed over the last twenty years (Figure 16).

A significant body of literature within the reviewed sample contributes to the thematic area of Predictive Maintenance in Industry 4.0, revealing a multidimensional and technologically rich research stream. These studies collectively emphasize the integration of artificial intelligence, machine learning, Internet of Things (IoT), and digital twin technologies to support advanced, data-driven maintenance strategies. Many works in this cluster highlight the transition from traditional condition-based monitoring toward more intelligent, predictive, and even proactive maintenance frameworks. For instance, the development of hybrid learning models for lithium-ion batteries [162], integration of predictive algorithms in composite smart structures [163], and the use of model-based systems engineering in mining maintenance [164] illustrate the sector-specific implementations of such approaches.

Moreover, publications increasingly link predictive maintenance to Industry 5.0 principles, such as human-centricity and system adaptability. For example, vision transformers integrated with digital twins are explored in the context of human-in-the-loop maintenance systems [165], while behavioral aspects of predictive maintenance are examined through a work system lens [20]. The role of AIoT communication and digital twin synchronization, as well as IoT, is illustrated in bridge maintenance applications [166,167], while resilient pharmaceutical manufacturing is addressed through digital twin-driven strategies in uncertain environments [168].

Further contributions include conceptual frameworks and applied models that leverage predictive maintenance in the context of Industry 4.0. These range from general strategic models [131,169] to scenario-specific approaches focusing on sustainability [170] and resilience-driven methodologies [171]. Together, this group of publications reflects a research trend toward predictive maintenance as a foundational element of digital transformation in industrial systems, aligning operational goals with sustainability, resilience, and human–system collaboration.

Taking one step further, a significant thematic cluster within the analyzed body of literature is centered on fault detection and diagnostics, closely linked to condition-based maintenance (CBM) approaches. This body of research focuses on the development of methods and technologies that enable the early identification of equipment faults, degradation patterns, and anomalies in various industrial environments. The growing adoption of artificial intelligence and advanced machine learning models has notably enhanced the predictive and diagnostic capabilities of modern maintenance systems.

Several studies explore hybrid or ensemble AI frameworks that integrate neural networks, genetic algorithms, and ensemble methods to improve the accuracy and robustness of diagnostic models, particularly in complex or novel applications, such as reinforced concrete structures [172]. Others focus on visual analytics for fault detection in manufacturing lines [173], transportation systems [174], or develop health indicators and pattern recognition methods to optimize diagnostics in smart manufacturing environments [175].

The role of digital twins is also prominent in CBM applications. They are used to support real-time fault monitoring and asset diagnostics in transportation systems, such as railway maintenance [129,176]. These studies illustrate how data fusion and digital models can support the lifecycle management of complex systems, including RL-enhanced strategies for infrastructure reliability [177].

Research on intelligent monitoring systems also shows significant progress, particularly in robotics and machinery. Monitoring of multi-axis robots [178] and fault-tolerant synchronization in multi-robot systems [179] are key topics reflecting Industry 5.0’s increasing emphasis on collaborative robotics. Similarly, deep learning techniques have proven effective for diagnostics in data-scarce or resource-constrained environments [180], and CNN-based methods are applied for defect detection in photovoltaic panels [181].

Moreover, studies such as the one by Zhang et al. [116] offer comprehensive overviews of prognostics and health management (PHM), highlighting the integration of AI for both real-time and long-term failure prediction across engineering systems. Other authors stress the importance of CBM in critical infrastructure, promoting it as a robust and sustainable strategy to enhance system resilience and operational continuity [182].

Collectively, the reviewed literature in this thematic cluster reveals a strong interdisciplinary focus on combining AI, sensor data, and simulation technologies to push the boundaries of fault detection, with applications spanning transportation, energy, robotics, smart manufacturing, and critical infrastructure.

The third thematic area that emerged strongly in the reviewed literature is human-centric maintenance, aligning closely with the principles of Industry 5.0. This research stream highlights the increasing recognition of human roles in smart maintenance environments, focusing on safety, well-being, decision empowerment, and collaborative interaction with intelligent systems.

Several studies propose frameworks and mechanisms that actively involve operators in decision loops and system adaptations. For example, refs. [183,184] outline development guidelines that ensure maintenance tasks are aligned with operator capabilities and sustainable goals. Under crisis conditions, refs. [126,136] demonstrate how explainable AI can empower workers with transparent recommendations. An artificial intelligence-based human-centric decision support framework under pandemic environments was proposed in [126], whereas [136] illustrates how human-centric AI can be used to enhance resilience, flexibility, and transparency in decision-making processes.

Studies such as [141,151] identify organizational and cognitive barriers, advocating for participatory design and continuous feedback. Real-time control strategies are exemplified by [185], while [128,186] showcase augmented reality interfaces that reduce cognitive load and enhance situational awareness.

In addition, a significant body of research also explores digital twin technologies with a human-oriented perspective, particularly in enhancing operators’ skills, safety, and productivity. The use of digital twins for operator training and safety receives attention in works [187,188,189]. Indeed, refs. [187,189] propose simulation models and frameworks that replicate human capabilities and interactions within digital manufacturing ecosystems.

Ergonomics and safety are also central to this thematic area. Papers such as [190,191] address physical and cognitive ergonomics in maintenance and operational tasks, utilizing sensors, computer vision, and data-driven safety models. In addition, the authors in [192] highlight how sensor data can be translated into actionable insights.

Lastly, human-centricity is embedded in broader conceptual frameworks that link digitalization and sustainable manufacturing. For example, refs. [183,193,194] explore how human values, competencies, and needs should shape the design of future maintenance and service systems. In addition, works [191,193] integrate ergonomic, safety, and sustainability considerations into end-to-end maintenance workflows. Together, these works illustrate a concerted shift toward socio-technical systems in which humans are not only beneficiaries of technology but active partners in maintaining and evolving industrial assets.

This body of literature reflects an ongoing shift from technology-centric paradigms toward integrated, socio-technical systems where human expertise, collaboration, and well-being are central design elements.

The thematic area of sustainable management of assets and environmental impact reflects a convergence of efforts to align maintenance practices with broader environmental and resilience goals. One of the key research directions involves the development of maintenance frameworks that incorporate sustainability indicators and resilience classifications. For instance, models supporting policy integration and multi-criteria decision-making contribute to aligning maintenance with sustainable development goals [195]. Likewise, the integration of standardized performance frameworks, such as EN 15341:2019, has been proposed to structure sustainable maintenance assessments [96].

Another prominent stream of research emphasizes the interplay between circular manufacturing systems and maintenance planning. Studies have highlighted how machine learning and intelligent design approaches can support sustainable operations through predictive analytics and the optimization of resource use [196]. This is complemented by work on robust layout planning using Big Data, which supports efficient facility configuration while reducing environmental footprint [197].

Environmental considerations in remanufacturing systems have also been explored through integrated production and maintenance models that consider uncertainty and degradation of components [198]. At the same time, specific sectors such as hydropower offer conceptual frameworks to assess the sustainability of maintenance management practices [199].

Emerging technologies play a central role in this thematic area. The adoption of secure digital infrastructures, such as zero-trust network-based access control systems, supports data integrity and environmental efficiency within Industry 4.0 [200]. Additionally, the application of machine learning in environmental monitoring is helping industries improve their operational resilience and environmental awareness, particularly in energy-intensive processes [201]. On the other hand, in [202] the authors focused on the impact of Maintenance 4.0 on sustainable development in the context of three dimensions: economy, environment, and risk.

The literature also reflects a strategic shift toward transformative resilience, where maintenance is embedded in adaptive strategies for sustainable industrial systems. This perspective positions resilience not just as risk mitigation, but as a catalyst for innovation and long-term ecological balance [203]. Furthermore, a case study from the automotive sector illustrates how Maintenance 4.0 approaches can form the foundation of sustainable manufacturing policies [204].

Finally, the integration of Industry 5.0 principles into product–service systems demonstrates a shift toward lifecycle thinking and the co-creation of sustainable value through intelligent maintenance planning [205]. These diverse contributions collectively underscore a dynamic and interdisciplinary research field, where asset management is increasingly positioned as a key enabler of environmental sustainability and system resilience.

It is also worth mentioning that a growing body of literature addresses the multifaceted challenge of enhancing resilience in both physical systems and supply chain networks, particularly under the transformative pressures of Industry 4.0 and 5.0. One key research avenue involves the integration of digital twin frameworks to anticipate disruptions and strengthen operational continuity. This includes proposals for digital twin–based support in the recovery and resilience of supply chains [206], as well as models enhancing factory resilience and optimization in future-oriented production environments [207]. Advanced decision-making tools are also being developed, such as a decision support framework that balances resilience and sustainability in service design [208], and a multi-criteria optimization approach for resilience-based maintenance, leveraging knapsack methods [74].

Supply chain-specific resilience has been further explored through AI-driven transformation strategies in Industry 5.0 [209], and predictive models for anticipating inbound logistics disruptions in volatile environments [210]. Several studies emphasize digital twins as enablers of sustainable and resilient manufacturing networks [211] and propose frameworks that merge blockchain with cognitive analytics to ensure robustness in additive manufacturing supply chains [212]. Blockchain technologies have also been considered essential to transforming supply chain management in Industry 5.0 for innovation and sustainability [213].

Beyond manufacturing and logistics, resilience is also considered in sector-specific contexts. Offshore wind turbine maintenance is addressed through a new risk management model emphasizing proactive resilience [214], while port expressways are studied for vehicle traffic resilience via lane management strategies [215]. Similarly, resilience in cooling tower operations has been optimized using data-driven process analysis [216], and in fleet systems, the evaluation of critical equipment maintenance strategies has been advanced [38].

The role of organizational dynamics is highlighted by research exploring robustness and resilience as catalysts for innovation in smart service factories [217], and frameworks that connect product–service system design with automated guided vehicles to promote resilient performance [218]. Capitalizing on AI capabilities to foster proactive adaptation to disruptive events is also being explored [219], along with multi-criteria decision approaches for improving global supply chain performance through Industry 4.0 integration [220].

The conceptual development of resilience assessment methods continues to evolve, including fuzzy-logic-supported maintenance strategies within the resilience-based maintenance paradigm [6] and systemic resilience assessment–driven maintenance strategy definition [7]. Altogether, these studies demonstrate the broad and interdisciplinary nature of resilience enhancement, encompassing digital modeling, intelligent systems, logistics optimization, and proactive risk management across diverse industrial and infrastructure contexts.

Finally, the last thematic area concerning maintenance-related concepts reflects a broad and multidimensional research scope that explores evolving approaches to managing maintenance in the context of digital transformation, sustainability, and resilience. Several studies highlight the theoretical and practical challenges in implementing next-generation maintenance paradigms such as Maintenance 5.0, particularly in balancing human involvement, automation, and sustainability goals [29]. For instance, the concept of adversarial maintenance has been proposed as a way to safeguard reliability and robustness in cyber-physical environments [78], while other works stress the role of Industry 5.0 principles in developing sustainable reliability-centered maintenance strategies [14].

The integration of new technologies is another major stream of research. Studies propose the application of augmented reality in facilitating maintenance operations [221], as well as intelligent software sensing in predictive and preventive strategies for IoT systems [222]. Maintenance optimization strategies in the context of Industry 4.0 are also widely explored, focusing on improving resource allocation, minimizing downtime, and enabling condition-based approaches [223]. Complementary to this, lean principles are analyzed in synergy with digital technologies to enhance sustainability in maintenance management [224].

A notable research direction is the integration of resilience into maintenance models and frameworks. Examples include resilient maintenance optimization for offshore wind turbines [214], port expressway operations [215], and complex buildings [171]. The resilience of safety management systems in building maintenance has also been conceptually addressed [225], and further developed in the context of plant process safety and sustainability [226].

From a systems perspective, some contributions focus on task scheduling and workforce coordination in dynamic maintenance environments [227,228], while others explore multi-dimensional integration, such as combining total productive maintenance with Industry 4.0 technologies for achieving global sustainability goals [229]. The remanufacturing of lithium-ion batteries as a sustainable maintenance practice within the Industry 5.0 paradigm is also examined [230].

Finally, the role of proactive and transformative strategies is evident in multiple works that emphasize future-oriented frameworks, aligning maintenance with long-term resilience, circular economy, and sustainability visions [203,231].

To sum up, the content-based analysis of the reviewed literature revealed a diverse and evolving landscape of research related to resilience-based maintenance (RBM) in the context of Industry 4.0 and 5.0. Six major thematic areas were identified.

Research on predictive maintenance focuses on integrating AI, IoT, and digital twins to anticipate failures, optimize maintenance actions, and enhance asset performance, particularly in complex or safety-critical infrastructures. Fault detection and diagnostics/CBM studies address advanced monitoring techniques, health indicators, and data fusion methods that support condition-based strategies in real-time environments. The human-centric maintenance domain highlights the increasing importance of operator engagement, ergonomics, human–machine interaction, and digital tools that support human-in-the-loop decision-making within sustainable and resilient manufacturing systems.

A substantial body of literature explores sustainable asset management, linking maintenance practices with environmental concerns, circular economy strategies, and the use of big data or machine learning to improve the sustainability and robustness of industrial operations. The area of systems and supply chain resilience emphasizes frameworks for managing disruptions, improving logistics and service design, and integrating technologies like digital twins, AI, and blockchain to foster adaptive capabilities. Finally, publications categorized under maintenance-related concepts address a wide range of emerging issues such as Maintenance 5.0, augmented reality applications, optimization algorithms, and conceptual frameworks for proactive and resilient strategies across sectors.

Together, these thematic areas underline the multidimensional nature of RBM and its alignment with digitalization, human factors, sustainability, and operational robustness. In the following section, a focused discussion is conducted based on the formulated research questions to identify key research challenges and gaps within this domain.

## 5. Discussion: Insights and Research Gaps

The reviewed literature provides a comprehensive view of the current landscape of resilience-based maintenance (RBM) and its evolution within the broader contexts of Industry 4.0 and Industry 5.0. Through the thematic classification of 93 publications, several key insights can be drawn regarding the state of research, employed technologies, alignment with emerging paradigms, and existing research gaps. Unlike earlier reviews that focused on isolated areas, such as predictive maintenance, AI diagnostics, or sustainability, this study offers a more integrative perspective by systematically linking resilience, sustainability, and human-centricity in maintenance systems.

In response to RQ1, the current state of RBM research reflects a mature but still fragmented field that is rapidly evolving. The six thematic areas identified, indeed, predictive maintenance, fault detection and diagnostics/CBM, human-centric maintenance, sustainable asset management, systems and supply chain resilience, and emerging maintenance-related concepts, demonstrate the growing multidimensionality of maintenance science. This review also deliberately adopts a cross-sectoral perspective, encompassing both industrial systems and infrastructure systems, which enhances the generalizability of the insights.

Although many studies provide advanced technical solutions or conceptual frameworks, few adopt a holistic, system-level view of resilience. There is a clear need for integrative models that align operational, strategic, and environmental resilience objectives across asset lifecycles. The present review addresses this gap by identifying critical intersections between resilience theory and maintenance practices, and by proposing a structured basis for developing future RBM frameworks that align with Industry 5.0 principles.

The concept of resilience-based maintenance (RBM) has gained considerable attention over the last decade, emerging at the intersection of asset management, risk engineering, and systems resilience. The literature indicates that RBM is not a singular methodology but rather an evolving paradigm shift in maintenance thinking, transitioning from reactive and predictive strategies to holistic approaches that account for adaptability, robustness, recoverability, and long-term operational continuity.

In industrial and infrastructure systems, resilience is increasingly being framed as a strategic requirement, not only for handling disruptions (e.g., failures, supply chain shocks, cyber threats), but also for sustaining performance under uncertainty. A wide array of studies conceptualize resilience from technical, organizational, and systemic perspectives, with growing interest in how maintenance contributes to system-wide resilience capacity.

Research to date shows that RBM is still relatively fragmented. Many studies introduce resilience as an auxiliary concept, often linked to condition-based maintenance, redundancy, or risk-based inspection, but lack a comprehensive integration of resilience indicators or structured evaluation frameworks. For instance, while fault-tolerant design and robust diagnostics are frequently referenced, the literature rarely operationalizes resilience using clear metrics (e.g., time to recovery, system degradation tolerance, performance under stress).

Nevertheless, there is a noticeable convergence of RBM with digital technologies, especially digital twins, simulation modeling, and AI-based decision support systems. These tools are increasingly used to model the behavior of systems under disturbances, optimize maintenance schedules based on risk and vulnerability profiles, and test recovery strategies in virtual environments. Several publications also point to multi-criteria decision-making methods (e.g., AHP, fuzzy logic, GINA, MICMAC) as mechanisms to support resilient maintenance planning, particularly in complex or safety-critical sectors.

Additionally, sector-specific research in transport, energy, water infrastructure, and manufacturing reflects tailored applications of RBM principles. Case studies highlight its relevance in railway systems, offshore wind turbine maintenance, cooling tower operations, and smart factories, often emphasizing the integration of resilience into both physical assets and cyber-physical systems.

In summary, the current state of RBM research reveals a rich but developing field, where the conceptual foundations are in place, but methodological consistency, implementation strategies, and measurement frameworks are still lacking. There is a strong emphasis on resilience as a qualitative or scenario-based objective, but fewer empirical studies provide validated models that can guide practitioners in designing, monitoring, and improving resilient maintenance strategies. Bridging this gap remains a key challenge and opportunity for future work in both academic and industrial contexts.

In addressing RQ2, artificial intelligence has become a cornerstone of RBM strategies. A wide range of methods, including neural networks, genetic algorithms, ensemble models, deep learning, reinforcement learning, and fuzzy logic, are deployed across use cases such as condition monitoring, predictive analytics, diagnostics, fault detection, and decision support. These tools are increasingly integrated with IoT, digital twins, and edge computing, enhancing real-time responsiveness and adaptive capacity. However, interoperability, scalability, and the quality of input data remain challenges in AI-enabled RBM systems, particularly in legacy industrial infrastructures or small and medium-sized enterprises (SMEs).

Taking a broader view, artificial intelligence (AI) plays a central role in enabling resilience-based maintenance (RBM) by enhancing the system’s ability to sense, interpret, adapt, and respond to uncertainties in dynamic industrial and infrastructure environments. The reviewed literature reveals a diverse landscape of AI techniques that are increasingly embedded into maintenance strategies to support decision-making, improve diagnostics, and enable learning mechanisms. Among the most prevalent AI methods used in RBM are machine learning (ML) algorithms, particularly supervised learning techniques such as decision trees, support vector machines (SVMs), random forests, and ensemble models. These methods are widely employed for fault classification, predictive diagnostics, and prognostics, often utilizing time-series data from sensors (e.g., vibration, temperature, acoustic signals). For instance, hybrid learning frameworks have been developed to support predictive maintenance of batteries, smart structures, and robotics systems, enhancing system adaptability to evolving conditions.

Deep learning (DL), including convolutional neural networks (CNNs) and recurrent neural networks (RNNs), has been increasingly applied in resource-constrained or data-scarce environments to detect complex patterns or anomalies. Applications include fault detection in photovoltaic panels, robotic arm diagnostics, and visual analytics in production lines. These models offer high performance in environments where resilience requires rapid and accurate detection of failure modes.

In the context of learning and adaptability, reinforcement learning (RL) and its deep variants (DRL) have emerged as promising tools for optimizing maintenance policies in stochastic or non-stationary environments. RL-based models enable systems to autonomously learn optimal maintenance actions by interacting with their environment, adjusting to changes in degradation rates or operating conditions. One study even integrates RL with lifecycle management strategies to support sustainable infrastructure resilience.

In addition, explainable AI (XAI) tools are gaining traction, especially in human-centric maintenance applications. These tools not only support technical resilience but also empower human operators by offering interpretable recommendations and traceable decision logic. This is particularly important under uncertain conditions (e.g., pandemics, cyber threats), where transparency enhances trust and collaboration between humans and AI systems.

The use of fuzzy logic, Bayesian networks, and multi-criteria decision-making (MCDM) approaches (e.g., AHP, TOPSIS, GINA) is also significant in RBM. These methods handle uncertainty and imprecise data, supporting decision processes where risk, system vulnerability, or resilience trade-offs must be evaluated. Such approaches are essential for integrating subjective expert knowledge and heterogeneous data sources into maintenance planning.

Moreover, AI is often coupled with digital twin (DT) platforms, enabling dynamic modeling of system behavior and virtual testing of maintenance scenarios. AI-enhanced digital twins allow for real-time monitoring, failure prediction, and resilience evaluation, thus fostering proactive interventions and simulation-based decision support.

To summarize, RBM research demonstrates an evolving and multidisciplinary application of AI, where tools are selected and tailored according to the complexity, criticality, and resilience goals of the system. Future developments are likely to focus on integrating adaptive AI models, real-time learning capabilities, and AI–human collaboration interfaces, paving the way for robust and intelligent maintenance ecosystems that sustain operational performance amid increasing uncertainty. The summary of techniques applied in RBM is given in Table 8.

With regard to the alignment of resilience-based maintenance with Industry 5.0 pillars (RQ3), a noticeable trend in the literature is the alignment of RBM with the pillars of Industry 5.0, especially human-centricity and sustainability. Human-in-the-loop systems, operator-centered interfaces, ergonomic risk reduction, and the use of AR/VR for decision support highlight how maintenance is becoming more people-oriented. At the same time, frameworks integrating circular economy principles, low-emission strategies, and sustainable production objectives demonstrate how RBM is being positioned as a driver of environmental and social responsibility. Still, many solutions remain in the conceptual or prototype phase, and their practical implementation across sectors requires further validation and standardization.

To better understand how resilience is operationalized in AI-driven maintenance contexts, the extracted metrics from reviewed studies were categorized according to their functional role. First, Table 9 presents a functional classification of resilience-related metrics based on the primary objective of measurement. The largest share of studies focused on performance and reliability indicators, such as availability, OEE, and MTBF, reflecting the field’s strong roots in reliability engineering.

Several papers applied predictive or prognostic metrics like RUL or health indicators, often in tandem with machine learning models. Others explored recovery-oriented metrics (e.g., MTTR, recovery delay), especially in contexts involving digital twins or supply chain disturbances. While a few studies incorporated structural or systemic resilience (e.g., survivability indices or supply chain robustness), the use of composite resilience metrics, which aggregate multiple resilience dimensions, remains relatively limited. Additionally, human-centric or organizational capacity indicators appear in emerging approaches, especially those aligned with the principles of Industry 5.0.

This classification underscores a growing interest in moving beyond failure prediction to more comprehensive assessments of resilience and adaptability within maintenance strategies. In addition, the detailed analysis of operational resilience metrics used in the reviewed papers is given in Appendix A.3, Table A3.

Second, to enhance interpretability, the identified sustainability-related metrics were functionally categorized according to their primary measurement purpose (Table 10). The most prevalent category includes energy-related indicators, such as energy consumption and cost efficiency (identified in 7 studies). This reflects the strong connection between maintenance operations and energy use optimization, especially in industrial environments where predictive strategies can minimize unnecessary power draw or support energy recovery.

A second group comprises emission-focused metrics, primarily related to CO_2_ and greenhouse gases (GHGs). Despite their relevance in sustainability discourse, such metrics are underrepresented across the sample. Even less frequent are circularity indicators, such as material reuse or recycling performance, which only appear in a handful of cases.

Some studies adopted a more integrated sustainability approach, applying multi-criteria decision analysis (MCDA) or composite indexes to evaluate trade-offs between performance, cost, and environmental impact. However, the overall scarcity of composite and standardized sustainability indicators points to a fragmented and non-uniform operationalization of sustainability in predictive maintenance contexts. In addition, the detailed analysis of sustainability KPI’s used in the reviewed papers is given in Appendix A.3, Table A4.

Despite growing recognition of sustainability as a key pillar of Industry 5.0, the integration of environmental and circularity-related indicators into resilience-based maintenance (RBM) frameworks remains limited. Our review reveals that only a minority of studies explicitly report CO_2_ emissions, energy consumption, or circular economy metrics, indicating a significant gap between theory and operational implementation.

Several barriers hinder the wider adoption of sustainability KPIs in RBM practice and research:

- Lack of standardization: There is no universally accepted framework for measuring sustainability performance within maintenance processes. Indicators such as carbon footprint or energy efficiency are defined and calculated differently across sectors, making comparison and benchmarking difficult.
- Data availability and granularity: Many maintenance systems are not yet equipped with sensors or data pipelines that enable real-time or historical tracking of energy use or emissions at a sufficiently granular level (e.g., per asset or maintenance action).
- Limited integration between maintenance and sustainability functions: In industrial settings, maintenance and sustainability are often managed by separate departments with different priorities. As a result, cross-functional KPIs are rarely developed or monitored jointly.
- Perceived misalignment with short-term objectives: Organizations may prioritize immediate operational metrics such as uptime or cost reduction, viewing sustainability metrics as secondary or long-term considerations.
- Tool and model immaturity: While digital twins, LCA (life cycle assessment) tools, and energy-aware predictive maintenance systems are emerging, they are still at early stages of maturity and not widely adopted.

Addressing these challenges requires both technical and organizational innovations. From a technical perspective, the development of standardized KPI sets, as well as AI-based tools capable of estimating indirect sustainability impacts of maintenance actions, is needed. Organizationally, closer integration between maintenance planning and environmental management strategies is essential to achieve Industry 5.0 goals.

On the other hand, one of the strongest points of convergence lies in sustainability. Numerous studies emphasize that RBM enables resource optimization, extends asset life cycles, and reduces environmental footprint by anticipating failures and supporting proactive interventions. Through predictive and condition-based strategies, RBM reduces unnecessary maintenance activities, spare part consumption, and energy-intensive repairs. Integration with circular manufacturing concepts, as seen in works applying machine learning for sustainable operations or robust layout planning, illustrates how maintenance functions are reoriented to support both economic and ecological resilience. Moreover, RBM is increasingly embedded in multi-criteria sustainability frameworks that consider environmental, social, and economic trade-offs in decision-making.

Moreover, RBM is increasingly embedded in multi-criteria sustainability frameworks that consider environmental, social, and economic trade-offs in decision-making.

In this context, energy-based maintenance (EBM) has recently emerged as a distinctive and complementary paradigm focused on optimizing maintenance interventions through continuous analysis of energy performance. Rather than treating energy consumption as a secondary operational metric, EBM positions energy data at the core of maintenance strategies, enabling the detection of inefficiencies, degradation trends, and energy-related anomalies in real time [104,233]. This paradigm aligns closely with the goals of sustainable maintenance, offering tangible benefits such as lower energy costs, reduced emissions, and improved system performance. Notably, recent contributions by Orošnjak et al. [105] have demonstrated how EBM can support cleaner production strategies by linking functional-productiveness with real-time energy indicators. As such, EBM reinforces the shift from purely reactive or reliability-centered strategies toward holistic approaches that integrate ecological awareness, data-driven prognostics, and resource efficiency in line with Industry 5.0 principles.

The human-centric pillar of Industry 5.0 is also strongly reflected in RBM literature. RBM systems are designed not only to adapt to technical disturbances but also to empower human operators and maintenance personnel. Studies on human-in-the-loop (HITL) frameworks, AR-enabled interfaces, ergonomic safety, and digital twins tailored for operator training demonstrate how RBM fosters collaboration between people and intelligent systems. Human–machine interaction is no longer incidental but essential, with humans acting as active participants in adaptive maintenance loops. For example, explainable AI (XAI) is used to make decisions interpretable, reducing cognitive overload and enhancing trust in automated recommendations.

However, as the role of human operators in AI-driven maintenance systems expands, new regulatory and safety challenges emerge. The integration of AI into maintenance decisions, particularly in systems involving human-in-the-loop (HITL) control, requires clear standards for accountability, transparency, and ethical oversight. Although frameworks such as the EU AI Act [148] and ISO/IEC 42001 [147] introduce principles for trustworthy AI, their practical implementation in industrial maintenance remains limited and often ambiguous. For instance, defining liability in partially autonomous maintenance decisions or ensuring that explainable AI (XAI) recommendations are truly understandable for frontline technicians poses both technical and governance challenges.

Moreover, there is currently no harmonized regulatory framework addressing how worker safety, psychosocial risks, or cognitive overload should be mitigated in AI-augmented maintenance environments [149]. As emphasized by Khurram et al. [149], the absence of sector-specific guidelines for human-centric AI in maintenance leaves a gap between conceptual aspirations and operational safety. This highlights the urgent need for interpretable, safety-compliant AI tools, co-designed with human factors experts and aligned with occupational health and ethical standards. Without such alignment, there is a risk that AI-enabled maintenance systems may inadvertently increase stress, deskill workers, or create ambiguous decision boundaries in critical operations.

Additionally, resilience in RBM is interpreted not only as a system’s capacity to recover from disruptions but also as a means of organizational learning and continuous improvement. This resonates with Industry 5.0’s vision of socio-technical ecosystems that are adaptable, inclusive, and oriented toward long-term value. Conceptual models of RBM include feedback loops, learning mechanisms, and resilience indicators that align with human competencies and organizational agility. Moreover, the systemic inclusion of ethics, worker safety, and mental well-being into maintenance planning signals a deepening of human-centric principles.

In sum, RBM is no longer a purely engineering-driven discipline but a strategic function that integrates technological intelligence, environmental stewardship, and human collaboration. The reviewed literature supports a growing consensus that RBM, as envisioned within the Industry 5.0 framework, is a key enabler of transformative resilience, creating value not only through operational continuity but also through ethical, sustainable, and participatory industrial practices. This transformation will, however, require not only technological innovation but also regulatory clarity, particularly in the areas of AI safety, operator accountability, and human rights compliance in automated decision-making contexts.

Finally, in response to RQ4, several research gaps and future directions have emerged. Despite the growing body of literature on resilience-based maintenance (RBM), several key research gaps and challenges remain that limit its widespread and effective implementation across industrial and infrastructure systems. These gaps span theoretical, methodological, technological, and practical dimensions, pointing to promising directions for future investigations.

One major challenge is the fragmentation of RBM conceptualizations. While resilience is often invoked in maintenance contexts, its interpretation varies widely, ranging from recovery time after failure to adaptability of systems to robustness under uncertainty. There is a lack of unified frameworks that integrate resilience with reliability, sustainability, and asset management principles in a consistent way. Furthermore, resilience indicators and metrics remain underdeveloped, particularly those that can quantify both technical and organizational aspects of RBM in real-time systems.

Another significant gap concerns the integration of human and organizational factors into RBM. Although Industry 5.0 emphasizes human-centricity, relatively few studies provide concrete models for incorporating human behavior, decision-making variability, or cognitive load into resilience strategies. Future research should focus on human-in-the-loop models, participatory approaches, and ergonomics-informed RBM frameworks that consider both physical and mental demands on operators and maintenance staff.

In addition, psychological and organizational resistance presents a critical barrier to the operationalization of RBM frameworks, particularly in the context of AI adoption. Employee trust, perceived loss of control, and fear of job displacement may limit acceptance of AI-enabled maintenance systems, despite their potential benefits [149]. Moreover, cultural inertia, lack of change readiness, and misalignment between technological innovation and organizational values can further hinder successful deployment. To ensure the long-term success of RBM, future studies should integrate perspectives from organizational psychology and change management, emphasizing interventions that foster engagement, trust, and transparent communication. As noted by Khurram et al. [149], AI solutions that fail to account for psychological safety, ethical concerns, and worker empowerment may undermine rather than enhance resilience in industrial settings. Therefore, research should move beyond the purely technical view and adopt more holistic approaches that address these socio-organizational dimensions. As a result, there is a growing need for interdisciplinary frameworks that combine technical modeling with organizational behaviour, change management, and communication strategies, especially in human-centric Industry 5.0 contexts.

In parallel, emerging regulatory and safety challenges present a critical research and implementation gap in the operationalization of human-centric RBM. As AI systems increasingly support or automate maintenance decisions, questions of transparency, accountability, and safety gain importance. Current regulatory initiatives, such as the EU AI Act [148] and ISO/IEC 42001 [147], offer high-level guidelines for trustworthy and ethical AI, but their application to the specific context of maintenance engineering remains underdeveloped. There is insufficient clarity on how to assign responsibility for AI-generated recommendations, how to validate explainability standards in operational settings, and how to ensure that AI tools respect human autonomy, especially in safety-critical infrastructure. These gaps create uncertainty among stakeholders and may further fuel organizational resistance, especially if workers perceive a lack of control, transparency, or recourse. Future research should address these questions through interdisciplinary approaches combining AI ethics, safety engineering, and legal frameworks, while also incorporating insights from human factors and cognitive ergonomics to ensure that AI-enabled RBM systems genuinely support, not undermine, operator trust, competence, and well-being.

Methodologically, limited work has been carried out on cross-sector validation of RBM frameworks. Most case studies are sector-specific, often focusing on energy, manufacturing, or transportation. Comparative studies across industries and infrastructure domains are needed to develop generalizable insights and modular RBM architectures. Additionally, RBM in small- and medium-sized enterprises (SMEs) remains underexplored, especially regarding scalability, cost-effectiveness, and access to AI and sensor technologies.

From a technological standpoint, while there is progress in using AI, digital twins, and IoT, the challenge lies in real-time data fusion, interoperability of digital ecosystems, and cybersecurity in RBM applications. Research should address how to handle incomplete or noisy data in resilience decision-making, how to ensure ethical use of AI in maintenance, and how to build trust in autonomous decision-support systems. Moreover, despite the promising potential of AI techniques, such as reinforcement learning, within resilience-based maintenance (RBM) frameworks, most of the current literature remains largely conceptual or descriptive. There is a notable lack of quantitative performance metrics, benchmarking studies, and critical assessments of the limitations of these AI approaches. This gap hinders a thorough understanding of their practical effectiveness and reliability in real-world maintenance scenarios. Consequently, it is essential to advance empirical research that rigorously evaluates AI-driven RBM methods using standardized metrics and comparative analyses. Such efforts would provide clearer insights into the trade-offs, scalability, and robustness of AI applications under varying industrial conditions. As a result, addressing these challenges will be crucial to transitioning from theoretical potential to validated, deployable solutions that can reliably enhance maintenance resilience and operational continuity. Until then, caution is advised in interpreting the current AI-related claims, and further interdisciplinary collaboration is needed to bridge the gap between AI research and maintenance engineering practice.

Finally, future studies should focus on developing maturity models, such as resilience or maintenance maturity frameworks, that help organizations benchmark their current capabilities and guide RBM implementation over time. Such models can be enriched by using multi-criteria decision-making, fuzzy logic, or Bayesian networks to deal with uncertainty and dynamic operational environments.

In summary, while RBM has made considerable advances, its further development demands a transdisciplinary approach, combining systems engineering, organizational psychology, sustainability science, and data science. Future work should prioritize building adaptive, explainable, and inclusive RBM systems that align with the complex demands of Industry 5.0.

To conclude, the current body of research reflects strong momentum toward transforming maintenance into a resilient, sustainable, and intelligent function of industrial systems. Nevertheless, to fully operationalize RBM within the frameworks of Industry 4.0 and 5.0, future research should address system-level integration, practical deployment, human-technology co-evolution, and long-term environmental impacts.

## 6. Implications and Future Outlook

Based on the findings of the conducted analyses and the synthesized insights from the discussion section, this study proposes a novel framework, the integrated maintenance maturity model (IMMM), to support organizations in assessing and enhancing their readiness for implementing resilience-based maintenance (RBM) strategies, particularly within the evolving context of Industry 5.0 and the adoption of AI-enabled technologies. The IMMM, developed in parallel with this systematic review, represents a multi-dimensional approach to addressing operational continuity and managing uncertainty, offering a practical lens through which maintenance practices can be evaluated and strategically advanced.

**Figure 17:**
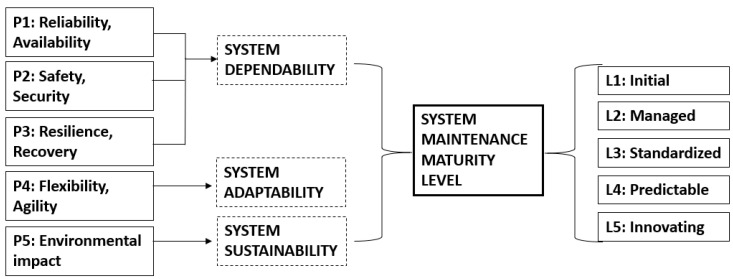
Proposed integrated maintenance maturity model (IMMM) framework. Source: own contribution.

The model, which will be fully detailed in a forthcoming publication titled “Multidimensional maintenance maturity modeling: predictive model and a case study on ensuring operational continuity under uncertainty” [234], builds upon the conceptual foundations laid out in this review. It translates theoretical constructs into actionable insights by organizing maintenance capabilities into five maintenance maturity potentials (P1–P5): reliability and availability, safety and security, resilience and recovery, flexibility and agility, and sustainability. These five dimensions correspond to broader system-level maturity dimensions, namely dependability (focusing on operational reliability and availability), adaptability (addressing agility and resilience in the face of disruptions), and sustainability (highlighting long-term ecological, resource, and ESG considerations). This mapping is illustrated in Figure 17, which is recommended to remain in the manuscript as a critical visual synthesis of the proposed approach.

Grounded in the conceptual layering, which delineates the barriers and enablers of maintenance performance across operational, resilience, and sustainability layers, the IMMM acts as a bridge between theory and practice. It offers a structured maturity roadmap that allows practitioners and decision-makers to identify their current positioning and plan progressive steps toward higher levels of maintenance excellence. The model incorporates a five-level maturity scale, ranging from Level 1 (initial) to Level 5 (innovating). Comprehensive descriptions of each potential area are provided in Table 11, whereas a detailed definition of maturity scale is presented in Table 12, Together, these elements facilitate targeted diagnostics and enable strategic prioritization of interventions.

In practice, the IMMM offers several strategic implications. First, it allows organizations to detect maturity gaps that may compromise resilience, especially under conditions of uncertainty, disruption, or regulatory pressure related to ESG standards. Second, it provides a structured way to prioritize investments in advanced maintenance technologies such as predictive analytics, AI-driven diagnostics, and energy-aware systems. Third, it aligns operational improvements with broader organizational goals by linking technical advancements in maintenance with long-term resilience and sustainability objectives. Furthermore, the framework is applicable as a diagnostic instrument in pilot studies or longitudinal evaluations, offering benchmarking capabilities across different industries and operational contexts.

From a research perspective, the development of the IMMM opens multiple avenues for further exploration. Empirical validation across industrial sectors, such as manufacturing, energy, and transportation, is essential to assess its predictive relevance and practical usability. Integration with digital twin platforms and computerized maintenance management systems (CMMSs) is anticipated to enable real-time tracking of maturity levels and facilitate dynamic decision-making. Moreover, the scoring methodology could be expanded through the use of fuzzy logic, allowing the model to incorporate expert judgment and handle uncertainty in data-scarce environments. Interdisciplinary extensions toward resilience engineering and cyber-physical systems design are also envisioned, especially in smart factory contexts where AI systems and human-in-the-loop approaches are co-evolving. Additionally, the development of sector-specific readiness benchmarks may support policymakers and certifying bodies in evaluating organizational preparedness for resilient and sustainable maintenance.

To further operationalize the IMMM, ongoing research is focused on developing a decision-support system that will integrate scenario modeling and maturity forecasting capabilities, offering organizations a dynamic tool for long-term maintenance planning and strategy formulation.

## 7. Conclusions

This article presented a systematic literature review on resilience-based maintenance (RBM) in the context of emerging digital and intelligent technologies. Drawing on 93 peer-reviewed publications from 2015 to 2025, the study synthesized key concepts and methodological approaches at the intersection of resilience, sustainability, and human-centric maintenance. The main contribution lies in mapping six major thematic areas, including fault detection and diagnostics, predictive strategies aligned with Industry 4.0, sustainable asset management, resilience engineering, and AI-based decision support.

The findings indicate that RBM is gaining momentum as a comprehensive approach addressing both anticipated and unexpected disruptions in technical systems. AI methods such as machine learning, reinforcement learning, and hybrid reasoning are increasingly integrated with technologies like digital twins and IoT, enabling adaptive, human-informed maintenance strategies. The keyword and co-citation analyses confirm the interdisciplinary nature of RBM, highlighting a shift from reactive to proactive and learning-based maintenance paradigms.

Despite the substantial progress, several research gaps remain. The fragmentation of definitions and frameworks, the limited inclusion of real-time human feedback in adaptive systems, and the lack of standardized assessment methods for resilience maturity pose challenges for both theory development and industrial implementation. Moreover, while digital twins and AI are increasingly integrated into maintenance ecosystems, their full potential for learning-based, long-term resilience optimization is not yet fully realized.

This review underscores the critical role of RBM in supporting the transition toward Industry 5.0, where sustainability, human well-being, and intelligent decision-making converge. The continued evolution of this field will require stronger methodological integration, broader cross-sectoral applications, and greater attention to ethical, organizational, and environmental implications.

In addition, one methodological limitation of the present review is the lack of a calculated interrater agreement score during the screening process. Although the selection was performed collaboratively by two researchers, without statistical assessment of agreement, the potential for subjective bias cannot be completely ruled out. Future studies may benefit from involving independent reviewers and calculating agreement indices such as Cohen’s kappa to enhance objectivity and reproducibility.

Future research on resilience-based maintenance (RBM) should prioritize the development of unified conceptual and operational frameworks that integrate resilience, predictive strategies, and sustainability in a coherent and transferable manner. Despite increasing interest in RBM, the current landscape remains fragmented, lacking standardized metrics and consistent methodologies. A crucial research avenue lies in further advancing the role of humans in the maintenance loop, particularly in the context of cognitive resilience, decision support, and adaptive learning, by leveraging technologies such as augmented and virtual reality or wearable sensors. Moreover, integrating digital twins with learning-based systems, such as reinforcement learning, offers great promise for building self-evolving maintenance solutions capable of adapting to dynamic operational conditions. Finally, future work must address the often-overlooked trade-offs between resilience and sustainability, identifying models that can optimize short-term recovery without compromising long-term environmental goals.

Currently, the authors are working on the development of an integrated resilience-based maintenance (RBM) framework that consolidates the key thematic areas identified in the literature review, particularly human-centric design, AI-supported decision-making, and sustainability principles. The next stages of the research will focus on translating this conceptual model into practical applications through pilot implementations, expert-based validation, and case studies conducted in industrial and infrastructure contexts. This approach aims to bridge the gap between theory and practice, enabling the deployment of resilient and intelligent maintenance strategies that are aligned with the transformative goals of Industry 5.0.

## Figures and Tables

**Table 1:** Evolution of maintenance strategies: from reactive to Maintenance 5.0. Source: own contribution based on [3,49,60].

Generation	Main Paradigm	Objective	Key Technologies/Tools	Data Dependency	Role of Human Operator	Integration Aspects
**1st Gen**	Reactive (Breakdown)	Restore function after failure	Manual inspection, basic tools	Very low	Manual diagnosis and repair	Isolated maintenance process
**2nd Gen**	Preventive (Time-based)	Reduce unexpected failures	Maintenance schedules, usage logs	Low	Planner and executor	Linked to production planning
**3rd Gen**	Predictive/ Condition-based	Predict failures before they occur	Sensors, SCADA, diagnostics, RUL estimation	Moderate to high	Data interpreter, condition assessor	Integrated with condition monitoring systems
**4th Gen**	Smart Maintenance	Real-time, autonomous decision-making	IoT, AI/ML, edge/cloud analytics, digital twins, CMMS	High	Decision supervisor, system integrator	Embedded in cyber-physical production systems
**5th Gen**	Maintenance 5.0	Sustainable, resilient, human-centric	AI, knowledge graphs, human–AI collaboration, LCA tools, XR interfaces	Very high	Ethical co-designer, cognitive collaborator	Interdisciplinary and system-wide, resilience-focused

SCADA—supervisory control and data acquisition; RUL—remaining useful life; LCA—life cycle assessment; XR—extended reality (AR/VR).

**Table 2:** Comparison of RCM, PdM, RxM, and RBM. Source: own contribution based on [6,74].

Feature/Strategy	Reliability-Centered Maintenance (RCM)	Predictive Maintenance (PdM)	Prescriptive Maintenance (RxM)	Resilience-Based Maintenance (RBM)
**Primary Objective**	Failure prevention	Failure prediction	Autonomous, optimized maintenance decisions	System adaptability and recovery
**Analytical Focus**	Failure modes and criticality	Degradation patterns, sensor data	Decision-making, optimization, risk-cost-performance trade-offs	System behavior under uncertainty
**Risk Consideration**	Known risks	Partially known (data-driven) risks	Known + uncertain risks with real-time trade-offs	Known + unknown risks
**Data Usage**	Historical data, expert judgment	Real-time condition monitoring	Real-time data + simulations + reinforcement learning feedback	Multi-source data, simulation, feedback
**Role of AI**	Limited (e.g., FMEA support)	Prognostics, ML-based diagnostics	Reinforcement learning, optimization algorithms, AI planning	RL, digital twins, knowledge graphs
**Human-Centric Integration**	Minimal	Limited	Moderate (human-in-the-loop or autonomous decision support)	Strong (decision support, cognitive AI)
**Reaction to Unexpected Events**	Low	Moderate	High (autonomous adaptation and intervention)	High (adaptive, learning-based)
**Maintenance Action Type**	Prescriptive (expert-defined rules)	Predictive (failure forecasts)	Prescriptive (optimal actions and scheduling, often autonomous)	Adaptive and resilient (system-level flexibility)

**Table 3:** Key indicators of sustainable maintenance under the triple bottom line (TBL) framework. Source: own contribution based on [111,113].

Dimension	Category	Indicator	Unit/Type	Description
**Environ-mental**	Energy efficiency	Energy consumption per maintenance activity	kWh/task	Measures the energy used per maintenance intervention
	Material sustainability	Percentage of recycled/reused parts	%	Share of reused or recycled components in total replaced items
	Environmental risk	Number of environmentally harmful incidents	count/year	Tracks incidents causing environmental harm (e.g., spills, emissions)
	Emissions reduction	The carbon footprint of maintenance operations	kg CO_2_ eq./month	Estimates GHG emissions from maintenance-related activities
**Economic**	Cost-effectiveness	Total cost of ownership (TCO)	currency/unit	A sum of acquisition, maintenance, and disposal costs over the asset lifecycle
	Downtime minimization	Average unplanned downtime	hours/month	Measures operational losses due to unexpected maintenance needs
	Maintenance productivity	Mean time to repair (MTTR)	hours	Reflects the average time required to complete maintenance interventions
	Asset longevity	Asset life extension due to maintenance	% or years	Measures improvement in asset lifespan thanks to effective maintenance
**Social**	Worker well-being	Number of safety incidents during maintenance	incidents/year	Tracks injuries or accidents during maintenance tasks
	Ergonomics and workload	Physical/cognitive strain assessment (survey-based)	qualitative (Likert scale)	Subjective or assessed level of strain experienced by workers
	Competence development	Training hours per maintenance employee	hours/year	Measures annual training and upskilling efforts
	Human–machine collaboration	Adoption of ergonomic/assistive technologies (e.g., cobots, AR)	binar/% of tasks supported	Tracks implementation of human-assistive tech in daily maintenance operations

**Table 4:** Comparative overview of traditional, smart, and sustainable maintenance approaches. Source: own contribution based on [87,96,112].

Aspect	Traditional Maintenance	Smart Maintenance (4.0)	Sustainable Maintenance (5.0)
**Paradigm**	Reactive/preventive	Predictive/prescriptive	Human-centric, sustainable, and resilient
**Primary Goal**	Restore function	Optimize asset performance	Balance performance with sustainability and social impact
**Decision-making**	Human-driven, rule-based	Data-driven, algorithm-based	Context-aware, value-based, collaborative
**Technology Enablers**	Basic sensors, manual tools	IoT, AI, digital twins, AR	Integrated CPS, green analytics, worker-assistive tech
**Data Usage**	Limited or non-existent	Extensive, real-time	Real-time + LCA metrics, social impact data
**Environmental Focus**	Minimal	Efficiency-oriented	Lifecycle optimization, emission minimization
**Economic Perspective**	Short-term cost reduction	Asset efficiency, reduced downtime	Lifecycle cost optimization and circular economy
**Social Considerations**	Low (focus on output)	Medium (operator efficiency)	High (safety, training, inclusion, job satisfaction)
**Resilience Integration**	Absent	Indirect (redundancy, alerts)	Direct (resilience engineering, adaptability, human-in-the-loop)
**Role of Human**	Executor	Supervisor/monitor	Partner/collaborator in hybrid systems

**Table 5:** Human-centric elements in maintenance technologies. Source: own contribution based on [20,124,128].

Technology	Human-Centric Element	Example Application
**AR-based Diagnostics**	Reduced cognitive load	Step-by-step AR-guided pump inspection
**AI Decision Support**	Explainability, confidence rating	Predictive maintenance with user validation
**Digital Twin**	Visual feedback, intuitive interaction	Operator-controlled system simulations
**Exoskeletons/Cobots**	Physical support and safety	Assisting in heavy part replacement tasks
**VR-based Training**	Skill development and scenario rehearsal	Emergency repair simulations for new workers

**Table 6:** Human-centricity across maintenance generations (1.0–5.0). Source: own contribution based on [3,140].

Aspect	Maintenance 1.0	Maintenance 2.0	Maintenance 3.0	Maintenance 4.0	Maintenance 5.0
**Operator Role**	Manual execution of repairs	Schedule-based execution, low autonomy	Increasing involvement in diagnostics, still reactive	Role shifts toward data interpretation and system oversight	Active co-decision-maker; empowered, context-aware, and ergonomically supported
**Decision-Making Model**	Fully manual decisions post-failure	Based on rules and fixed intervals	Data-informed decisions with human supervision	AI-supported decisions with limited human feedback	Human-in-the-loop and human-on-the-loop frameworks fully integrated
**Human-Technology Interaction**	Tools only, no digital interface	Paper-based logs, basic CMMS	Use of sensors and dashboards	IoT interfaces, AR/VR systems, mobile apps	Seamless and personalized interfaces (wearables, XR, cognitive support)
**Safety and Ergonomics**	Minimal, reactive	Basic compliance-based ergonomics	Condition monitoring supports safety	Real-time alerts, digital twins for safe task execution	Proactive ergonomics, well-being analytics, worker co-designed systems
**Learning and Skills Development**	Learning through experience, manuals	Structured training programs	Training in digital tools, early simulations	Digital learning platforms, AR-based instruction	Continuous, AI-driven upskilling; personalized and inclusive learning pathways
**Ethics and Inclusion**	Not considered	Rarely addressed	Initial considerations in system design	Inclusion as a feature in HMI design	Core principle: equity, transparency, inclusion, and ethics embedded from design to operation
**Transition barriers**	**Transition barriers for → 2.0** - Lack of formal training and standards - Limited awareness of preventive maintenance benefits - Resistance to change from manual processes	**Transition barriers for → 3.0** - Inconsistent implementation of scheduled maintenance - Low autonomy causing inflexibility - Limited digital literacy among operators	**Transition barriers for → 4.0** - Fragmented adoption of sensor technology - Reactive culture still dominant - Data interpretation challenges - Lack of integration between systems	**Transition barriers for → 5.0** - Technical complexity of AI and IoT integration - Institutional resistance to change - Trust issues with AI decisions - High implementation costs - Need for cross-disciplinary collaboration	

**Table 7:** Inclusion and exclusion criteria used in the screening process.

Criterion Type	Inclusion Criteria	Exclusion Criteria
**Language**	Articles published in English	Non-English publications
**Publication type**	Peer-reviewed journal articles	Review papers, book chapters, editorials, conference papers, theses
**Subject area**	Engineering, manufacturing, operations, maintenance, and industrial systems	Medicine, biology, chemistry, social sciences unrelated to industrial systems
**Time period**	Published between 2015 and 2025	Outside of this range
**Topical relevance**	Addresses RBM, predictive maintenance, sustainable/circular maintenance, human-centricity in industrial contexts, or smart technologies in maintenance	Articles that do not address any of the core concepts of resilience, predictive, or sustainable maintenance
**Methodological quality**	Presents original research with clear objectives, rigorous methodology, and contributions to theory or practice	Lacks methodological rigor or conceptual relevance
**Full-text availability**	Full-text available and accessible	Abstract-only or inaccessible full texts

**Table 8:** AI techniques applied in resilience-based maintenance (RBM).

AI Technique	Application in RBM	Benefits of RBM	Example Studies
**Machine Learning (ML)**	Predictive modeling of failures; estimation of remaining useful life (RUL)	Improves failure anticipation; supports dynamic maintenance planning	[162,196,201,223]
**Deep Learning (DL)**	Fault detection in complex systems; diagnostics based on image/signal data	High accuracy in anomaly detection; effective in unstructured data environments	[180,181,185]
**Reinforcement Learning (RL)**	Adaptive maintenance scheduling; autonomous decision-making in uncertain conditions	Enables learning-based optimization; adapts to changing operational contexts	[177,185,222]
**Fuzzy Logic**	Modeling expert knowledge; decision-making under uncertainty	Captures imprecise criteria; facilitates resilience modeling with linguistic variables	[6,7,78]
**Hybrid Models (e.g., ML + Fuzzy + RL)**	Robust diagnostics; multi-objective maintenance optimization	Combines interpretability and adaptability; enhances model performance in uncertain settings	[78,172,177,223]
**Explainable AI (XAI)**	Transparent support for maintenance decisions; human-centric diagnostics	Enhances trust; enables operator involvement; supports regulatory requirements	[126,136]
**Genetic Algorithms/Evolutionary Methods**	Optimization of maintenance scheduling and resource allocation	Efficient in complex search spaces; supports multi-criteria resilience planning	[74,172]
**Computer Vision/CNNs**	Visual inspection of assets; ergonomic and defect detection	Enables non-invasive diagnostics; enhances safety and reliability assessment	[173,181,190,191]
**Digital Twins (DTs) + AI**	Predictive simulation; dynamic decision-making and system modeling	Supports resilience scenarios; integrates real-time data for continuous system awareness	[165,166,168,187,207,211]
**AIoT (AI + Internet of Things)**	Autonomous diagnostics; condition monitoring in cyber-physical systems	Real-time data-driven insights; improves responsiveness and situational awareness	[167,201,219]

**Table 9:** Functional typology of operational resilience metrics used in reviewed studies.

Type of Metric	Example Indicators	Frequency	Ref.
**Predictive/Prognostic**	RUL, health indicator, SOC, SOH, residual criticality	7	[74]
**Recovery-related**	Time to recovery, delay, MTTR, completion time, recovery indicator	6	[166]
**Performance and Reliability**	Availability, OEE, MTBF, maintenance frequency, failure rate, service level, state-of-charge (SOC), state-of-health (SOH)	9	[7,38,129,162,198,200,202,204,222]
**Structural/SC Resilience**	Supply chain resilience, structural condition, survivability index, business continuity	6	[45,177,212,216,220,226]
**Composite/Resilience Indexes**	Resilience score, robustness, resilience level, influence coefficient	7	[7,12,13,38,197,208,211]
**Resource Efficiency/Sustainability**	Resilience-sustainability score, value-added time, budget constraints	3	[195,204,208]
**Organizational Capacity**	Maintenance support potential, operation time, service availability, task scheduling, access interruption	5	[6,189,200,222,227]
**Human-Centric/Contextual**	Feasibility of monitoring, human-in-the-loop reward, skills acquisition metrics	2	[14,185]
**Traffic/Flow Resilience**	Overall traffic capacity, traffic service monitoring	2	[215,222,232]

**Table 10:** Functional classification of sustainability KPI’s in maintenance and production systems.

Type of Sustainability Metric	Example Indicators	Frequency	Ref.
**Energy Efficiency and Consumption**	Energy consumption, energy efficiency, energy utilization rate, energy recycling rate, energy-related penalty, energy cost savings	7	[185,197,202,205,216,220,222]
**Emissions and Environmental Impact**	Greenhouse gas emissions, carbon consumption, carbon emission factors, average CO_2_ emissions, pollution, environmental monitoring services, environmental sustainability	7	[14,129,177,198,212,222,228]
**Material and Resource Use/Circularity**	Material utilization rate, material recycling rate, resource efficiency, 6R principles, renewable energy, waste minimization, waste disposal monitoring	6	[14,204,205,220,222,228]
**Sustainability Performance Indexes**	Sustainability metrics, green score, sustainability score, LEVEL(S) structure indicators, sustainability ROI metrics, environmental indicators	6	[14,195,204,208,220,226]
**Composite Trade-offs (with Resilience, etc.)**	Sustainability-resilience score, weighted ROI combining safety, sustainability, reliability, and resilience	3	[208,220,226]
**Smart Infrastructure and ICT-Based Metrics**	Public lighting monitoring, environment service monitoring, smart energy service levels, IoT-based control KPIs	2	[185,222]
**Safety and Risk-Aware Sustainability**	Economy-safety utility value, safety, and sustainability ROI	2	[177,226]

**Table 11:** Maintenance maturity potentials in the developed approach.

Potentials	Main Purpose	Knowledge Area	Measurement Area	Main Indicators	Measurement Purpose	Intended Outcome
**P1: Reliability, availability**	Uninterrupted operation	**Reliability Engineering**	System reliability and operational continuity	- Mean time between failures (MTBF) - Uptime percentage - Failure rate	- To assess failure frequency and impact on operations - To ensure system availability and minimize downtimes	- Improved system reliability - Reduced unexpected downtimes - Increased equipment lifespan
**P2: Safety, security**	Protection against threats	**Safety and Security Engineering**	Operational safety and response effectiveness	- Safety incident rate - Incident frequency - Response time to threats	- To monitor and reduce safety risks - To improve response strategies and mitigate potential hazards	- Reduced number of safety incidents - Faster response to security threats - Improved workplace safety
**P3: Resilience, recovery**	Renewable resilience	**Resilience Engineering**	System recovery capability and failure impact	- Recovery time objective (RTO) - Time to recover after a failure - System downtime after incidents	- To evaluate the system’s ability to recover from disruptions - To minimize downtime and ensure operational continuity	- Faster recovery from failures - Reduced downtime impact on operations - Increased system robustness
**P4: Flexibility, agility**	Short-term adaptation	**Control Engineering**	Adaptability to changes	- Response time to change - Agility index - Time to adapt to internal/external changes	- To assess how quickly the system can respond to changes - To enhance adaptability in dynamic environments	- Improved responsiveness to market and operational changes - Increased operational efficiency - Better risk management
**P5: Environmental impact**	Long-term environmental friendliness	**Environmental Engineering and Sustainability**	Environmental impact and resource efficiency	- Carbon footprint - Resource efficiency - Waste reduction percentage	- To reduce environmental impact and optimize resource use - To support sustainable and responsible operations	- Lower CO_2_ emissions - Increased energy and resource efficiency - Minimized waste generation

**Table 12:** Maintenance maturity matrix in the developed approach.

Li\Pi	P1: Reliability, Availability	P2: Safety, Security	P3: Resilience, Recovery	P4: Flexibility, Agility	P5: Sustainability
**L1: Initial**	Failures are logged, but no predictive or preventive measures exist. Downtime tracking is inconsistent.	Safety incidents are logged ad hoc, with no systematic analysis or response strategies.	Recovery times are inconsistent, with no clear RTOs or contingency plans. Systems often experience prolonged downtime after incidents.	Changes are addressed reactively, leading to inefficiencies and delays; processes are slow and often reactive.	Environmental impact is not systematically tracked, and no formal sustainability initiatives exist.
**L2: Managed**	Regular maintenance stabilizes uptime, MTBF is tracked at a basic level, and failure rates are analyzed post-mortem.	Some safety protocols are established, but there are inconsistencies in implementation across units; response times to threats vary significantly.	Recovery protocols are established for local units, but recovery times are still unpredictable; RTOs are loosely defined.	Basic adaptability measures exist, but response times are inconsistent across different units.	Some environmental initiatives exist, but sustainability efforts are not fully integrated into maintenance workflows.
**L3: Standardized**	Standard processes for preventive maintenance are established and utilized across all units, improving consistency in MTBF and reducing failure rates.	Safety procedures are standardized, with routine training, safety incident reporting, and structured risk mitigation.	Recovery procedures are standardized, with defined RTOs and downtime reduction strategies in place.	Standardized processes improve response times to operational changes, but adaptation is still slow in unpredictable conditions.	Sustainability goals (e.g., carbon footprint reduction, waste management) are integrated into maintenance practices, with measurable goals.
**L4: Predictable**	Downtime events are statistically analyzed, predictive maintenance models are developed, and real-time failure trends are monitored.	Data analysis proactively manages safety risks, leading to faster response times and reduced incident frequency.	Recovery strategies are optimized based on statistical analysis, ensuring predictable RTOs and minimal disruptions.	Processes are dynamically adjusted based on statistical models, improving response times and flexibility.	Sustainability metrics (e.g., resource efficiency, CO_2_ reduction) are actively tracked, with consistent improvements in resource efficiency and waste reduction.
**L5: Innovating**	Proactive reliability improvement programs using real-time data analytics, AI-driven predictive maintenance, and reliability optimization to reduce failures and maximize system availability.	Advanced safety technologies (e.g., AI-based threat detection) continuously improve security and risk mitigation.	Continuous improvement of recovery strategies, integrating real-time monitoring and analysis to reduce RTO and downtime and enhance system resilience.	Highly flexible systems capable of rapid adaptation, with continuous feedback loops to optimize response to changes. Self-optimizing processes dynamically adjust based on AI-driven analytics, ensuring rapid adaptation.	Innovative sustainability practices are continuously implemented, focusing on achieving long-term environmental goals and reducing the organization’s carbon footprint.

## Supplementary Materials

The following supporting information can be downloaded at https://www.mdpi.com/article/10.3390/s25165100/s1, Table S1: The PRISMA Checklist.

## Abbreviations

The following abbreviations are used in this manuscript:

**Abbreviation**	**Full Description**
AHP	Analytical Hierarchy Process
AI	Artificial Intelligence
AR	Augmented Reality
CBM	Condition-Based Maintenance
CM	Condition Monitoring
CMMSs	Computerized Maintenance Management Systems
CNNs	Convolutional Neural Networks
CPS	Cyber-physical System
CR	Corrective Maintenance
DL	Deep Learning
DRL	Deep Reinforcement Learning
DT	Digital Twin
EBM	Energy-based Maintenance
ERP	Enterprise Resource Planning
GHG	Greenhouse Gases
GINA	Grey Influence Analysis
HITL	Human-in-the-loop
HMI	Human Machine Interface
HSM	Health Status Monitoring
IoT	Internet of Things
ISM	Interpretive Structural Modeling
KPI	Key Performance Indicator
LCA	Life Cycle Assessment
LCC	Life Cycle Costing
MCDM	Multi-criteria Decision-making
MICMAC	Matrix of Cross-Impact Multiplications Applied to Classification
ML	Machine Learning
MTTR	Mean Time To Repair
O&M	Operation and Maintenance
PdM	Predictive Maintenance
PHM	Prognostics and Health Management
PM	Preventive Maintenance
PRISMA	Preferred Reporting Items for Systematic Reviews and Meta-Analyses
RBM	Resilience-based Maintenance
RCM	Reliability Centered Maintenance
RL	Reinforcement Learning
RNNs	Recurrent Neural Networks
RUL	Residual Lifetime
RxM	Prescriptive Maintenance
SCADA	Supervisory Control and Data Acquisition
SLR	Systematic Literature Review
SMEs	Small and Medium Enterprises
SVMs	Support Vector Machines
TBL	Triple Bottom Line
TCO	Total Cost of Ownership
VR	Virtual Reality
XR	Extended Reality
WoS	Web of Science

## Appendix A

### Appendix A.1

**Table A1:** A summary of the recent papers focused on the literature overview on resilience-based, sustainable-based maintenance in relation to Industry 4.0/5.0.

Ref.	Publ. Year	Research Objectives	Maintenance Focus	Scope/Industry Context	Review Type	Identified Gaps
[235]	2018	To explore enabling technologies for Operator 4.0 and classify them by functions and interaction modalities.	**Human-centric smart maintenance, operator support**	Cross-industry, Industry 4.0 environments	Technology-focused survey	Lack of integration between technologies; need for ergonomic and cognitive support in digitalized maintenance
[9]	2019	To analyze how sustainability principles are integrated into maintenance strategies in manufacturing.	**Sustainable manufacturing maintenance**	Industrial/manufacturing systems	Narrative review	Lack of integration between sustainability dimensions; limited frameworks aligning economic, environmental, and social aspects
[32]	2019	To explore how Maintenance 4.0 technologies support sustainable manufacturing by enhancing equipment longevity, reducing downtime, and addressing economic, environmental, and social dimensions of value creation.	**Maintenance 4.0 and Sustainability**	Sustainable manufacturing	Overview review	Lack of practical tools and digital maturity models
[17]	2020	To systematically review and classify predictive maintenance initiatives in Industry 4.0, highlighting methods, standards, and challenges while proposing a novel taxonomy to guide multidisciplinary research.	**Predictive maintenance methods and tools**	Industry 4.0	Systematic literature review	Does not address future shift toward Industry 5.0; lacks coverage of human and cyber dimensions
[236]	2021	To map and classify maintenance literature in Industry 4.0 from 2015 to 2020.	**Maintenance 4.0 adoption and AR technologies**	Manufacturing, smart factories (Industry 4.0)	Mapping review (systematic search + conceptual clustering)	Need for empirical validation; lack of human-centric integration; fragmentation among AR/IoT/ML solutions
[34]	2021	To identify trends in sustainable manufacturing technologies through a systematic literature review and develop a conceptual research framework.	**Sustainable Manufacturing**	Production, eco-innovation	Systematic review	Lack of integration of human-centric approach and workers’ experience
[10]	2022	To review sustainable maintenance strategies for single and multicomponent equipment.	**Sustainable strategies and implementation**	Equipment-level, multicomponent systems	Thematic review	Few integrative strategies; poor linkage to real-time condition monitoring and decision-making tools
[16]	2022	To review and categorize intelligent predictive maintenance models and workflows in Industry 4.0 and propose a decision-support platform to enhance smart maintenance practices.	**Predictive maintenance—models and technical challenges**	Industry 4.0	Systematic literature review	Little focus on human and sustainability dimensions; lacks Industry 5.0 alignment
[19]	2022	To conduct a systematic review of predictive maintenance challenges and propose a new classification framework, emphasizing its ongoing relevance in Industry 4.0 environments.	**Predictive Maintenance**	Industry 4.0, smart factories	Systematic literature review	Focused only on predictive maintenance in I4.0 environments
[42]	2022	To examine how digitalization enables sustainable maintenance practices.	**Sustainable maintenance via digital services**	Cross-industry maintenance services	Systematic literature review	Lack of integration between digital tools and sustainability metrics in maintenance
[45]	2023	To analyze maintenance models supporting reuse/remanufacturing using bibliometric and content review.	**Circular maintenance (reuse, remanufacturing)**	Sustainable manufacturing contexts	Umbrella review + bibliometric	Limited focus on AI-driven tools, predictive resilience, and Industry 5.0 readiness
[1]	2023	To develop an integrated Maintenance 5.0 framework that bridges traditional and advanced maintenance strategies by addressing sustainability, human-centricity, and the adoption of Industry 4.0 technologies, especially in SMEs.	**Transition from Maintenance 4.0 to 5.0 (human-centric, AI-driven, sustainable)**	Global manufacturing, focus on SMEs and zero-defect manufacturing	Systematic literature review	Lack of sustainability/environmental KPIs, limited integration of human factors, and missing transition paths for SMEs
[3]	2023	To systematically review and categorize key technological domains of Maintenance 4.0—AR/VR, system architecture, data-driven decisions, Operator 4.0, and cybersecurity—to identify research trends and gaps guiding future studies.	**Maintenance performance indicators in Industry 4.0 context**	Broad industrial context	Bibliometric + systematic review	Weak mapping of human-centric aspects
[20]	2023	To investigate human, task, and organizational factors affecting predictive maintenance systems’ acceptance and identify key enablers for successful PdM adoption through literature synthesis and expert interviews.	**Predictive Maintenance with a human-centric approach**	Human–machine collaboration in Industry 5.0	Conceptual review with empirical elements	Need for human-behaviour integration in maintenance planning and execution
[33]	2023	To review and analyze sustainable maintenance decision-making models, focusing on integrating economic, environmental, and social dimensions, and to identify research trends, gaps, and opportunities for developing implementable, data-driven solutions.	**Sustainability in Maintenance**	Modeling-based academic research	Systematic literature review	Limited integration of sustainability indicators into maintenance decision models
[237]	2023	To provide updated bibliometric mapping and thematic trends in Industry 5.0.	**I5.0 trends (incl. maintenance)**	Broad, cross-sector context	Bibliometric review using Scopus and Biblioshiny	Early stage of I5.0 research; lack of clarity in definition; limited maintenance-specific focus
[1]	2023	To synthesize literature on emerging Maintenance 5.0 concept and evolution of RBM.	**Emerging paradigm of Maintenance 5.0**	Industrial and infrastructure systems (inferred)	Systematic literature review	Integration of resilience, sustainability, and AI remains conceptual; lack of standardized metrics
[11]	2023	To review total productive maintenance (TPM) from a sustainability perspective.	**Sustainable TPM**	Cross-sectoral (industrial focus)	Systematic literature review	Scarce alignment between TPM and sustainability indicators; limited empirical validation of STPM models
[13]	2024	To identify key technology drivers enabling sustainable maintenance in manufacturing.	**Sustainable maintenance with focus on digital enablers**	Manufacturing industries	Technology-driven review	Insufficient evaluation of digital tools in sustainability metrics; lack of maturity models or implementation roadmaps
[12]	2024	To identify and synthesize criteria for adopting sustainable maintenance practices.	**Sustainable maintenance adoption criteria**	General/cross-sector	Umbrella review (review of reviews)	Fragmentation of approaches; lack of standardized indicators and strategic frameworks
[15]	2024	To develop a multi-layered framework integrating Industry 5.0 principles with predictive maintenance and condition monitoring to enhance sustainability, resilience, and human-centricity.	**Predictive maintenance and condition monitoring evolution**	Industry-wide + case study	Systematic review + case study	Weak attention to cybersecurity
[27]	2024	To compare maintenance strategies in Industry 4.0 and Industry 5.0, evaluate their technological, procedural, and human-centric shifts, and guide industrial stakeholders in adapting maintenance approaches for enhanced resilience and competitiveness.	**Comparative: I4.0 vs. I5.0 Maintenance**	Industrial evolution toward human-centric and sustainable systems	Comparative review	Lack of clear transition models, human-role redefinition, and socio-technical frameworks
[4]	2025	To review the evolution from Maintenance 4.0 to 5.0 by analyzing sustainability integration and human-centric challenges in the transition framework.	**Shift toward Maintenance 5.0, human and sustainability dimensions**	Cross-industry, Industry 5.0	Systematic literature review	Lack of integration of social and human dimensions; limited guidance for technological-human convergence
[46]	2025	To consolidate insights from reviews on sustainable maintenance and validate findings through a questionnaire-based survey.	**Sustainable maintenance; cleaner production**	Cross-sector with industrial relevance	Umbrella review + survey	Lack of integrative frameworks linking sustainability to digitalization and human-centricity
[51]	2025	To systematize the literature on prescriptive maintenance and identify future research avenues.	**Prescriptive maintenance and decision support**	Broad: cross-industry application	Systematic literature review	Absence of cross-sectoral benchmarks, low integration of sustainability criteria

### Appendix A.2

**Table A2:** Number of citations per selected papers according to Scopus database (papers with minimum one citation).

No.	Ref. Title	Source	Citation Number According to Scopus Database
1	A New Concept of Digital Twin Supporting Optimization and Resilience of Factories of the Future	Applied Sciences (Switzerland)	140
2	Artificial Intelligence in Prognostics and Health Management	Engineering Applications of Artificial Intelligence	107
3	Maintenance Optimization in Industry 4.0	Reliability Engineering and System Safety	107
4	Predictive Maintenance Planning for Industry 4.0: Framework and Case Study	Sustainability (Switzerland)	103
5	Sustainable Robust Layout using Big Data Approach	Journal of Cleaner Production	98
6	Artificial Intelligence-based Human-centric Decision Support Framework Under Pandemic Environments	Annals of Operations Research	60
7	Pattern Recognition Method of Fault Diagnostics for Smart manufacturing	Mechanical Systems and Signal Processing	53
8	AIoT-informed Digital Twin Communication for Bridge Maintenance	Automation in Construction	51
9	Predictive Maintenance for industry 5.0: Behavioural Inquiries from a Work System Perspective	International Journal of Production Research	49
10	A New Resilient Risk Management Model for Offshore Wind Turbine Maintenance	Safety Science	42
11	On Sustainable Predictive Maintenance: Exploration of Key Barriers	Sustainable Production and Consumption	37
12	Process Resilience Analysis based Data-Driven Maintenance Optimization: Cooling Tower Operations	Computers and Chemical Engineering	35
13	Resilience of Process Plant: What, Why, and How Resilience Can Improve Safety and Sustainability	Sustainability (Switzerland)	35
14	Human-oriented Maintenance and Disassembly in Sustainable Manufacturing	Computers and Industrial Engineering	33
15	Machine Learning Integrated Design for Resilient Circular Manufacturing Systems	Computers and Industrial Engineering	28
16	Leveraging Blockchain for Sustainability and Supply Chain Resilience in e-Commerce Channels for Additive Manufacturing: A Cognitive Analytics Management Framework-Based Assessment	Computers and Industrial Engineering	25
17	Digital Twins for Managing Railway Maintenance and Resilience	Open Research Europe	25
18	Environmental Issue in Integrated Production and Maintenance Control of Unreliable Manufacturing/Remanufacturing Systems	International Journal of Production Research	22
19	Condition-based Monitoring as a Robust Strategy towards Sustainable and Resilient Infrastructure	Sustainable and Resilient Infrastructure	22
20	Using Fuzzy Logic to Support Maintenance Decisions According to Resilience-Based Maintenance Concept	Eksploatacja i Niezawodnosc	21
21	Contribution of Maintenance 4.0 in Sustainable Development with an Industrial Case Study	Sustainability (Switzerland)	21
22	Sustainability of Maintenance Management Practices in Hydropower Plant	Materials Today: Proceedings	20
23	Developing Resilience for Safety Management Systems in Building Repair and Maintenance: A Conceptual Model	Safety Science	20
24	Integrating Industry 4.0 and Total Productive Maintenance for Global Sustainability	TQM Journal	20
25	Towards Human Digital Twins to Enhance Workers’ Safety and Production System Resilience	IFAC-PapersOnLine	18
26	A Human–Machine Interaction Mechanism: Additive Manufacturing for Industry 5.0—Design and Management	Sustainability (Switzerland)	18
27	AI-Driven Supply Chain Transformation in Industry 5.0: Enhancing Resilience and Sustainability	Journal of the Knowledge Economy	16
28	Machine Learning Aided Rail Corrugation Monitoring for Railway Track Maintenance	Structural Monitoring and Maintenance	15
29	Digital Twins in Condition-based Maintenance Apps: A Case Study for Train Axle Bearings	Computers in Industry	13
30	Challenges of Human-Centered Manufacturing in the Aspect of Industry 5.0 Assumptions	IFAC-PapersOnLine	13
31	XAI Sustainable Human in the Loop Maintenance	IFAC-PapersOnLine	13
32	Fast Augmented Reality Authoring: Fast Creation of AR Step-by-Step Procedures for Maintenance Operations	IEEE Access	12
33	Simulation-Based Digital Twins Enabling Smart Services for Machine Operations: An Industry 5.0 Approach	International Journal of Human-Computer Interaction	12
34	Fleet Resilience: Evaluating Maintenance Strategies in Critical Equipment	Applied Sciences (Switzerland)	11
35	Intelligent Monitoring of Multi-axis Robots for Online Diagnostics of Unknown Arm Deviations	Journal of Intelligent Manufacturing	11
36	A Zero-Trust Network-Based Access Control Scheme for Sustainable and Resilient Industry 5.0	IEEE Access	10
37	The Confluence of Digital Twin and Blockchain Technologies in Industry 5.0: Transforming Supply Chain Management for Innovation and Sustainability	Journal of the Knowledge Economy	10
38	Proposal of Industry 5.0-Enabled Sustainability of Product–Service Systems and Its Quantitative Multi-Criteria Decision-Making Method	Processes	10
39	A Fusion of Neural, Genetic and Ensemble Machine Learning Approaches for Engineering Predictive Capabilities	Powder Technology	9
40	Synergies between Lean and Industry 4.0 for Enhanced Maintenance Management in Sustainable Operations: A Model Proposal	Processes	9
41	Information Technologies in Complex Socio-technical Systems based on Functional Variability: A Case Study on HVAC Maintenance Work Orders	Applied Sciences (Switzerland)	7
42	Toward Sustainability and Resilience with Industry 4.0 and Industry 5.0	Frontiers in Manufacturing Technology	7
43	Integration of MBSE into Mining Industry: Predictive Maintenance System	International Journal of Emerging Technology and Advanced Engineering	6
44	Deep Learning based Approaches for Intelligent Industrial Machinery Health Management and Fault Diagnosis in Resource-Constrained Environments	Scientific Reports	6
45	A Conceptual Digital Twin Framework for Supply Chain Recovery and Resilience	Supply Chain Analytics	6
46	Human-in-the-Loop Control Strategy for Smart Manufacturing Using Fuzzy Control	Procedia Computer Science	5
47	Resilient Manufacturing Systems Enabled by AI Support to AR equipped operator	2021 IEEE ICE/ITMC	4
48	Maintenance Strategies Definition Based on Systemic Resilience Assessment: A Fuzzy Approach	Mathematics	4
49	A Human-centric Approach to Aid in Assessing Maintenance from the Sustainable Manufacturing Perspective	Procedia Computer Science	4
50	A resilience-based Maintenance Optimisation Framework using Multiple Criteria and Knapsack Methods	Reliability Engineering and System Safety	4
51	Analyzing the Role of Digital Twins in Developing a Resilient Sustainable Manufacturing Supply Chain: A Grey Influence Analysis (GINA) Approach	Technological Forecasting and Social Change	4
52	Integration of Inspection and Monitoring Data for RL-Enhanced Life-Cycle Management of Infrastructure Networks	Structure and Infrastructure Engineering	3
53	Resilient Design of Product Service Systems with Automated Guided Vehicles	Vehicles	3
54	Fostering Lithium-Ion Battery Remanufacturing through Industry 5.0	International Journal on Interactive Design and Manufacturing	2
55	A Framework for Integrating Vision Transformers with Digital Twins in Industry 5.0 Context	Machines	2
56	Hybrid Machine Learning Framework for Predictive Maintenance and Anomaly Detection in Lithium-Ion Batteries Using Enhanced Random Forest	Scientific Reports	2
57	How to Predict Disruptions in the Inbound Supply Chain in a Volatile Environment	Advances in Transdisciplinary Engineering	1
58	A Decision Support Framework for Resilient and Sustainable Service Design	Global Journal of Flexible Systems Management	1
59	Architecture for Fault Detection in Sandwich Panel Production Using Visual Analytics	Hybrid Artificial Intelligent Systems	1
60	Embracing Resilience in Pharmaceutical Manufacturing: “digital twins”—Forging a Resilient Path in the VUCA maze	International Journal of Pharmaceutical and Healthcare Marketing	1
61	Distributed Maintenance Task Scheduling for Multiple Technician Teams Considering Uncertain Durations and Deterioration Effects towards Industry 5.0	International Journal of Production Research	1
62	Guardians of Reliability, Robustness, and Resilience: Adversarial Maintenance in the Era of Industry 4.0 and 5.0	Procedia Computer Science	1
63	Validation of Computer Vision-based Ergonomic Risk Assessment Tools for Real Manufacturing Environments	Scientific Reports	1
64	Proactive Maintenance Strategy Based on Resilience Empowerment for Complex Buildings	Smart Innovation, Systems and Technologies	1

### Appendix A.3

**Table A3:** Operational resilience metrics used in the reviewed studies.

No.	Ref. Title	Ref.	Used Operational Resilience Metrics
2	Artificial Intelligence in Prognostics and Health Management	[116]	Residual lifetime (RUL) metrics
5	Sustainable Robust Layout using Big Data Approach	[197]	Robustness
7	Pattern Recognition Method of Fault Diagnostics for Smart Manufacturing	[175]	Health indicator
8	AIoT-informed Digital Twin Communication for Bridge Maintenance	[166]	Time delay
12	Process Resilience Analysis based Data-driven Maintenance Optimization: Cooling Tower Operations	[216]	Resilience survivability index
13	Resilience of Process Plant: What, Why, and How Resilience Can Improve Safety and Sustainability	[226]	System survivability index; business continuity
16	Leveraging Blockchain for Sustainability and Supply Chain Resilience in e-Commerce Channels for Additive Manufacturing: A Cognitive Analytics Management Framework-Based Assessment	[212]	Supply chain resilience
17	Digital Twins for Managing Railway Maintenance and Resilience	[129]	Maintenance frequency
18	Environmental Issue in an Integrated Production and Maintenance Control of Unreliable Manufacturing/Remanufacturing Systems	[198]	Expected/average number of failures
20	Using Fuzzy Logic to Support Maintenance Decisions According to Resilience-based Maintenance Concept	[6]	Organization’s maintenance support potential level;
21	Contribution of Maintenance 4.0 in Sustainable Development with an Industrial Case Study	[202]	OEE
29	Digital Twins in Condition-based Maintenance Apps: A Case Study for Train Axle Bearings	[176]	RUL
34	Fleet resilience: evaluating maintenance strategies in critical equipment	[38]	System availability, systemic resilience index
35	Intelligent Monitoring of Multi-axis Robots for Online Diagnostics of Unknown Arm Deviations	[178]	Health indicator
36	A Zero-Trust Network-Based Access Control Scheme for Sustainable and Resilient Industry 5.0	[200]	Failure rate, access interrupt
40	Synergies between Lean and Industry 4.0 for Enhanced Maintenance Management in Sustainable Operations: A Model Proposal	[224]	MTTR; MTBF
45	A Conceptual Digital Twin Framework for Supply Chain Recovery and Resilience	[206]	Recovery indicator
48	Maintenance Strategies Definition Based on Systemic Resilience Assessment: A Fuzzy Approach	[7]	Resilience level, availability
49	A Human-centric Approach to Aid in Assessing Maintenance from the Sustainable Manufacturing Perspective	[183]	RUL scoring function
50	A Resilience-based Maintenance Optimisation Framework using Multiple Criteria and Knapsack Methods	[74]	System residual criticality
51	Analyzing the Role of Digital Twins in Developing a Resilient Sustainable Manufacturing Supply Chain: A Grey Influence Analysis (GINA) Approach	[211]	Total influence coefficient
52	Integration of Inspection and Monitoring Data for RL-Enhanced Life-Cycle Management of Infrastructure Networks	[177]	Structural condition
56	Hybrid Machine Learning Framework for Predictive Maintenance and Anomaly Detection in Lithium-Ion Batteries using Enhanced Random Forest	[162]	State-of-charge (SOC); state-of-health (SOH); RUL metric
58	A Decision Support Framework for Resilient and Sustainable Service Design	[208]	Resilience-sustainability score; expected implementation cost; available budget; number of resilience strategies
61	Distributed Maintenance Task Scheduling for Multiple Technician Teams Considering Uncertain Durations and Deterioration Effects towards Industry 5.0	[227]	Maintenance service level; completion time
64	Proactive Maintenance Strategy Based on Resilience Empowerment for Complex Buildings	[171]	Time to failure
73	Human-in-the-Loop Control Strategy for IoT-based Smart Thermostats with Deep Reinforcement Learning	[185]	Reward function
77	Towards Human-Centric Digital Simulation: Guidelines to Simulate Operators Skills Acquisition	[189]	Operation’s processing time
86	Resilience Maintenance Strategy for Mixed Vehicle Traffic on Port Expressway based on Lane Management	[215]	Overall traffic capacity
87	Maintenance and Asset Management Improvement Based on Resilience and Sustainability Classification Systems	[195]	Resilience dimensions and indicators
89	A Systematic Approach of Maintenance 4.0 Towards a Sustainable Manufacturing Policy: A Case Study on an Automobile Company	[204]	OEE index; value-added time
90	Smart Preventive Maintenance of Hybrid Networks and IoT Systems Using Software Sensing and Future State Prediction	[222]	Availability of services during the test period; traffic service levels monitoring
91	Industry 5.0 for Sustainable Reliability Centered Maintenance	[14]	RCM indicators, including R3: feasibility of condition monitoring; R4: operating conditions for health monitoring
92	Enhancing Sustainable Global Supply Chain Performance: A Multi-Criteria Decision-Making-Based Approach to Industry 4.0 and AI Integration	[220]	Supply chain resilience

**Table A4:** Sustainability KPI’s used in the reviewed studies.

No.	Ref. Title	Ref.	Sustainability KPI’s
5	Sustainable Robust Layout using Big Data Approach	[197]	Energy consumption
12	Process Resilience Analysis based Data-driven Maintenance Optimization: Cooling Tower Operations	[216]	Energy costs
13	Resilience of Process Plant: What, Why, and How Resilience Can Improve Safety and Sustainability	[226]	Sustainability weighted ROI metric; safety and sustainability ROI metric; Safety, sustainability, reliability, and resilience weighted ROI metric
16	Leveraging Blockchain for Sustainability and Supply Chain Resilience in e-Commerce Channels for Additive M: A Cognitive Analytics Management Framework-Based Assessment	[212]	Environmental sustainability
17	Digital Twins for Managing Railway Maintenance and Resilience	[129]	Greenhouse gas emissions; carbon emission factors
18	Environmental Issue in an Integrated production and Maintenance Control of Unreliable Manufacturing/Remanufacturing systems	[198]	Carbon consumption plan
21	Contribution of Maintenance 4.0 in Sustainable Development with an Industrial Case Study	[202]	Energy efficiency
38	Proposal of Industry 5.0-Enabled Sustainability of Product–Service Systems and Its Quantitative Multi-Criteria Decision-Making Method	[205]	Energy efficiency ratio; energy utilization rate; energy recycling rate; material utilization rate, material recycling rate
52	Integration of Inspection and Monitoring Data for RL-Enhanced Life-Cycle Management of Infrastructure Networks	[177]	Economy and safety single-attribute utility value; carbon emission
58	A Decision Support Framework for Resilient and Sustainable Service Design	[208]	Resilience-sustainability score
73	Human-in-the-loop control strategy for IoT-based smart thermostats with Deep Reinforcement Learning	[185]	Energy-related penalty
83	Task Scheduling Strategy for 3DPCP Considering Multidynamic Information Perturbation in Green Scene	[228]	Average CO_2_ emissions per unit of product quality
87	Maintenance and Asset Management Improvement Based on Resilience and Sustainability Classification Systems	[195]	The LEVEL(S) structure indicators
89	A Systematic Approach of Maintenance 4.0 Towards a Sustainable Manufacturing Policy: A Case Study on an Automobile Company	[204]	Sustainability metrics
90	Smart Preventive Maintenance of Hybrid Networks and IoT Systems Using Software Sensing and Future State Prediction	[222]	Energy distribution service level; environment monitoring service; waste disposal monitoring service; public lighting monitoring service
91	Industry 5.0 for Sustainable Reliability Centered Maintenance	[14]	SCM sustainability metrics, including: pollution; resource/energy consumption; 6R principles in service design; renewable energy
92	Enhancing Sustainable Global Supply Chain Performance: A Multi-Criteria Decision-Making-Based Approach to Industry 4.0 and AI Integration	[220]	Environmental indicators, e.g., emissions monitoring and reduction; energy efficiency; resource efficiency and waste minimization; circular economy integration
